# Structure-Guided Antiviral Peptides Identification Targeting the HIV-1 Integrase

**DOI:** 10.1021/acsphyschemau.4c00006

**Published:** 2024-07-05

**Authors:** Md. Shahadat Hossain, Md. Siddik Alom, Mohammad Salauddin Kader, Mohammed Akhter Hossain, Mohammad A. Halim

**Affiliations:** † Division of Infectious Diseases and Division of Computer Aided Drug Design, The Red-Green Research Centre, BICCB, 16 Tejkunipara, Tejgaon, Dhaka 1215, Bangladesh; ‡ Department of Pharmacy, Faculty of Life Science, Mawlana Bhashani Science & Technology University, Tangail 1902, Bangladesh; § Ohio State Biochemistry Program, The Ohio State University, Columbus, Ohio 43210, United States; ∥ Center for RNA Biology, The Ohio State University, Columbus, Ohio 43210, United States; ⊥ Department of Chemistry, Saint Louis University, Saint Louis, Missouri 63101, United States; # The Florey, University of Melbourne, Melbourne, Victoria 3010, Australia; ∇ Department of Chemistry and Biochemistry, Kennesaw State University, Kennesaw, Georgia 30144, United States

**Keywords:** HIV-1 integrase, peptide inhibitor, molecular dynamics, anti-HIV drugs, peptide−protein interaction

## Abstract

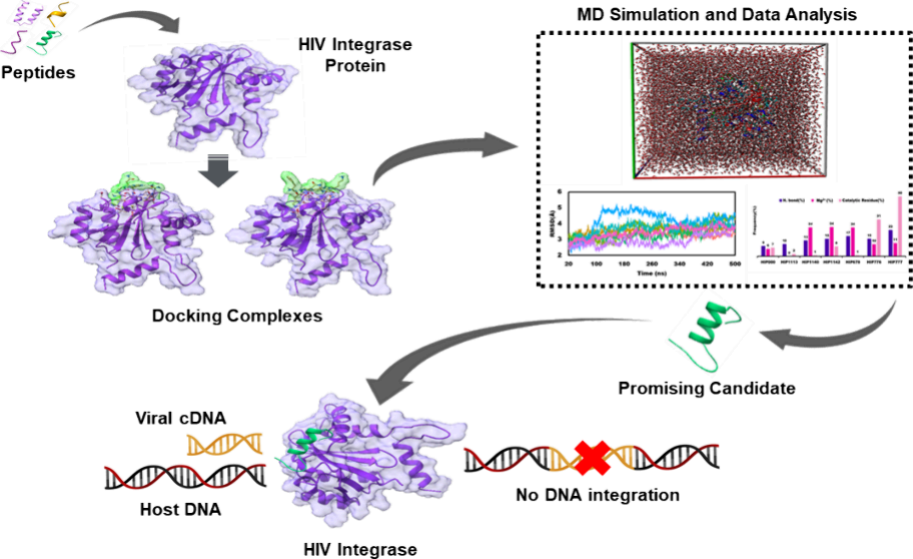

HIV-1 integrase (IN), a major protein in the HIV life cycle responsible for integrating viral cDNA into the host DNA, represents a promising drug target. Small peptides have emerged as antiviral therapeutics for HIV because of their facile synthesis, highly selective nature, and fewer side effects. However, selecting the best candidates from a vast pool of peptides is a daunting task. In this study, multistep virtual screening was employed to identify potential peptides from a list of 280 HIV inhibitory peptides. Initially, 80 peptides were selected based on their minimum inhibitory concentrations (MIC). Then, molecular docking was performed to evaluate their binding scores compared to HIP000 and HIP00N which are experimentally validated HIV-1 integrase binding peptides that were used as a positive and negative control, respectively. The top-scoring docked complexes, namely, IN-HIP1113, IN-HIP1140, IN-HIP1142, IN-HIP678, IN-HIP776, and IN-HIP777, were subjected to initial 500 ns molecular dynamics (MD) simulations. Subsequently, HIP776, HIP777, and HIP1142 were selected for an in-depth mechanistic study of peptide interactions, with multiple simulations conducted for each complex spanning one microsecond. Independent simulations of the peptides, along with comparisons to the bound state, were performed to elucidate the conformational dynamics of the peptides. These peptides exhibit strong interactions with specific residues, as revealed by snapshot interaction analysis. Notably, LYS159, LYS156, VAL150, and GLU69 residues are prominently involved in these interactions. Additionally, residue-based binding free energy (BFE) calculations highlight the significance of HIS67, GLN148, GLN146, and SER147 residues within the binding pocket. Furthermore, the structure–activity relationship (SAR) analysis demonstrated that aromatic amino acids and the overall volume of peptides are the two major contributors to the docking scores. The best peptides will be validated experimentally by incorporating SAR properties, aiming to develop them as therapeutic agents and structural models for future peptide-based HIV-1 drug design, addressing the urgent need for effective HIV treatments.

## Introduction

**Figure 1:**
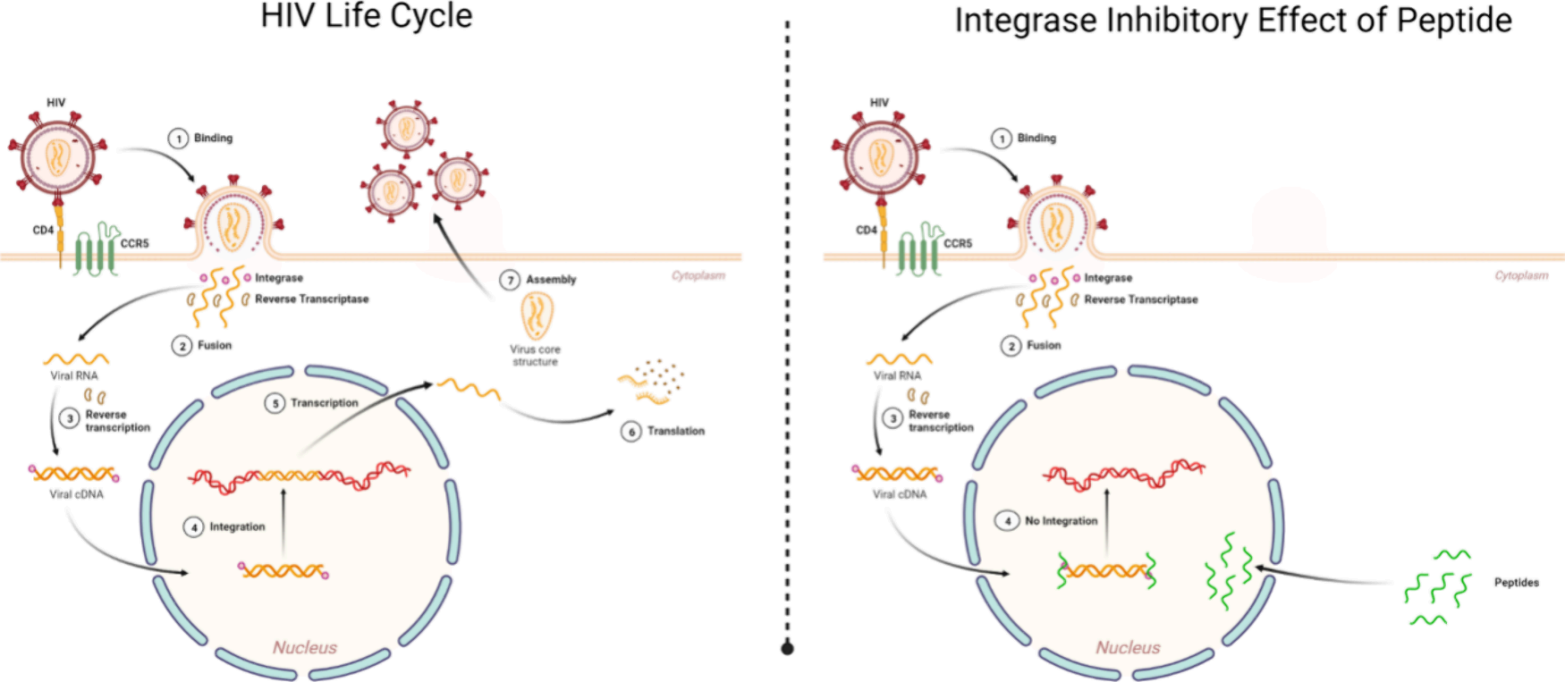
Role of integrase in HIV-1 life cycle and peptide inhibitor mechanism.

Human Immunodeficiency Virus-1 (HIV-1) infection continues to be a global public health issue, with millions of people living with HIV and thousands of new infections each year, mainly affecting the middle and lower economic world.^1,2^ According to the United Nations Program, in 2021, 38.4 million people are living with HIV, wherein around 650,000 people died from HIV-related diseases worldwide.^3^ HIV-1 is composed of several proteins that are essential for the viral life cycle. The envelope protein (gp120 and gp41),^4^ capsid protein (p24),^5^ protease (PR),^8^ reverse transcriptase (RT), and integrase (IN)^6,7^ are the main proteins of HIV-1 along with other proteins including Vif, Vpu, Vpr, and Nef.^8,9^ Among these viral proteins, HIV-1 Integrase is an essential enzyme for the replication of the virus and appears to be a vital target for drug/peptide design.^10^ During the biogenesis of viral particles, RT converts viral RNA into cDNA.^11^ The insertion of newly synthesized viral cDNA into the genome of the host cell is catalyzed by IN, a critical step in the viral life cycle (Figure 1).^10,12^ Integrase is a 32.0 kDa protein that is encoded by the HIV-1 integrase gene.^12,13^ It has three main domains: the N-terminal domain, the catalytic core domain, and the C-terminal domain.^13^ The enzymatic active site is located in the catalytic core domain;^12^ the N-terminal domain helps to recruit the viral DNA to the active site; and the C-terminal domain stabilizes the enzyme.^12^

There are several classes of antiretroviral drugs that are currently used to inhibit the activity of IN, including Integrase Strand Transfer Inhibitors (INSTIs).^14,15^ These drugs bind to the active site of integrase and prevent the transfer of viral cDNA into the host cell’s genome.^16^ Examples of INSTIs include raltegravir, elvitegravir, dolutegravir, bictegravir, cabotegravir.^14,16^ Raltegravir was the first FDA-approved INSTI, and it has been shown to be effective in suppressing the replication of the virus and slowing the progression of HIV-1 to AIDS.^14^ A newer INSTI candidate, known as Elvitegravir, is often used in combination with other antiretroviral drugs in a single-tablet regimen.^17^ Some studies showed that dolutegravir, another INSTI, has been very effective in suppressing the replication of the virus and slowing the progression of HIV-1 to AIDS.^14,18^ Recent studies revealed that Bictegravir, a novel INSTI, has been found to have high potency.^19,20^ Moreover, Cabotegravir is also a novel integrase inhibitor that is given as a long-acting injection.^21−23^ However, the key disadvantages of these drugs are: they need to be administered in combination with two or more drugs, a gain of weight, neuropsychiatric, etc.^14,24,25^

Although small molecule-based antiviral drugs targeting the HIV-1 integrase showed a lot of promise, drug resistance is a global concern, where continuing viral replication and extended drug exposure lead to resistant. In this regard, peptides have several advantages including stimulating low resistance, low toxicity, high specificity, effectiveness, easy of synthesis, high potency, fewer side effects, and low accumulation in tissue. Various early research has been conducted on the use of peptides as inhibitors of the enzyme,^26−30^ but it is still considered to be in the early stages of development. One of the main advantages of using peptides as inhibitors is their small in size and high specificity for the target enzyme,^31,32^ which may make them less likely to cause side effects compared to other small molecule inhibitors.^33^ There have been some studies that have reported promising results with peptide inhibitors of IN,^33^ and other similar studies used peptides as promising inhibitors.^34,35^ For instance, one study found that a peptide derived from the C-terminal domain of HIV-1 integrase was able to inhibit the activity of the enzyme in vitro.^36^ Another study identified a peptide that binds to the active site of integrase and prevents the transfer of viral DNA into the host cell’s genome.^37,38^ However, these studies are mostly in the preclinical stage, and more research is required to determine the safety and efficacy of peptide inhibitors in vivo, and to develop peptides that are effective enough to be used as therapeutics.

To elucidate the peptide interaction mechanism with the HIV integrase protein and provide insights for future structure-based drug design targeting HIV integrase, a structure-guided computational approach was employed. This approach was applied within the context of an experimental integrase inhibitory peptide library. Based on the computational screening and HADDOCK scores, the six best docked complexes, such as IN-HIP1113, IN-HIP1140, IN-HIP1142, IN-HIP678, IN-HIP776, and IN-HIP777, were selected. Subsequently, a 500 ns molecular dynamics (MD) simulation was conducted on these selected complexes. Among the six superior complexes, IN-HIP776, IN-HIP777, and HIP1142 uphold stable interactions over the simulation period and interact with the active residues of the HIV-1 integrase, indicating their ability to interfere with the catalytic activity of the enzyme. To delve deeper into the mechanism of action, extended simulations were conducted on these three selected peptides, alongside independent peptide simulations were also run to explore conformational variations. The structure–activity relationship (SAR) study showed that aromatic amino acids and the overall volume of the peptides are crucial for strong interactions. The results were thoroughly analyzed and compared with positive (HIP000) and negative (HIP00N) control peptides to enhance the robustness of the findings. Further experimental structural validation is required to confirm their inhibitory mechanism and determine their pharmacokinetic properties. Nevertheless, these peptides provide valuable insights and a solid foundation for the design and optimization of novel peptide inhibitors for the treatment of HIV-1 infection.

## Methods

### Database Selection and Primary Screening

This study focused on the use of the HIV inhibitory peptides database (HIPdb)^39^ to identify the best peptides and their inhibitory mechanism against the integrase protein of HIV that can effectively inhibit HIV-1. The database contains 981 potential peptides against HIV, among which there are 280 that are specific to HIV-1 integrase. These peptides were screened based on their inhibitory concentrations listed on the database (μM vs nM range), and 80 of the most potent peptides were selected for molecular docking study.

### Peptide Modeling and Molecular Docking

**Figure 2:**
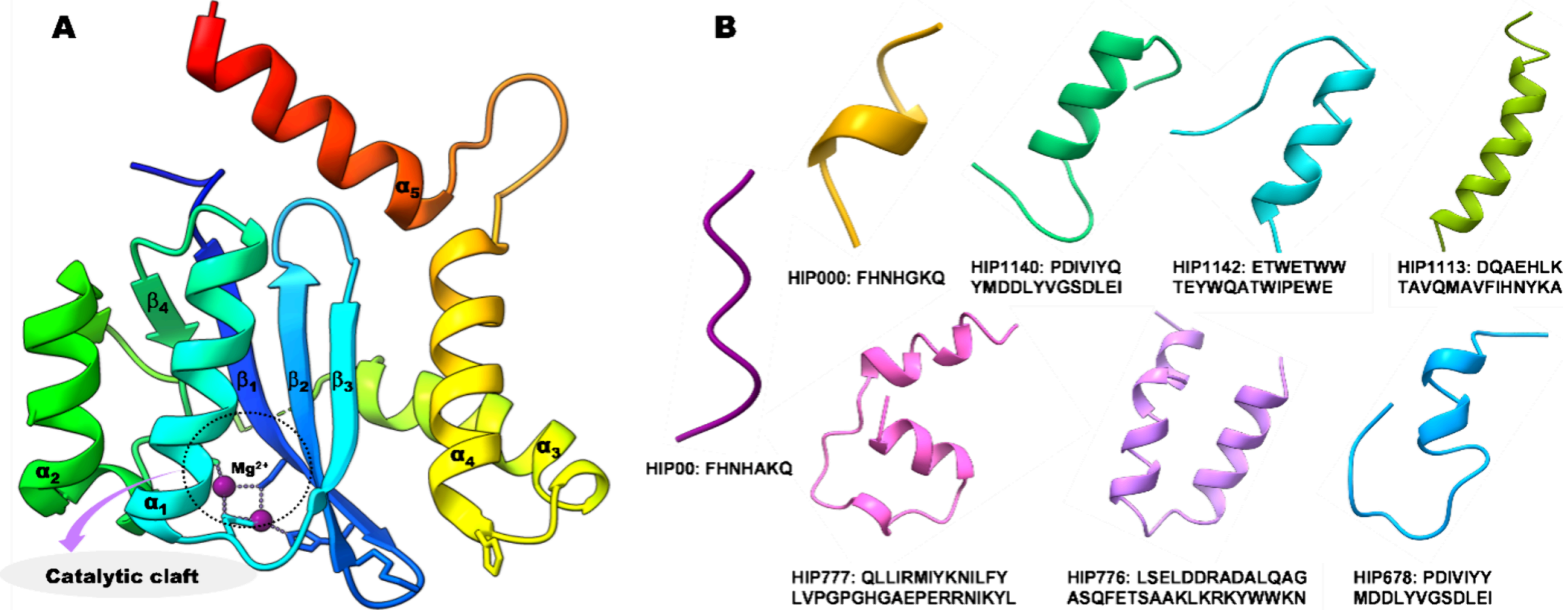
Protein and peptide structural information. (A) Structural properties of the catalytic core domain of HIV-1 integrase protein (B) Structure of top-six peptides along with the control peptides and their corresponding amino acid sequences. Peptides were modeled using PEP-FOLD.^40^ To better understand the structural properties and potential binding interactions of these selected peptides, a comprehensive figure showcasing the 3D views of integrase protein (Figure 2A) with its appropriate structural information and the best six peptides with the positive (HIP000) and negative (HIP00N) control peptides (Figure 2B) have been presented. The structures of these peptides have been color-coded for easy identification, and their corresponding amino acid sequences are prominently displayed alongside the 3D representations.

The selected 80 peptides with high integrase inhibitory properties were modeled using PEP-FOLD.^40^ The crystal structure of the HIV-1 integrase was obtained from the Protein Data Bank (PDB ID: 1EXQ).^13^ Water and hetero atoms were removed except for two divalent Mg^2+^ ions. Missing residues were assigned using SWISS-MODEL^41^ with the crystal structure as a template (PDB ID: 1EXQ(13)). The peptides were then docked to the substrate binding sites of the HIV-1 integrase using PatchDock^42^ and the top 1000 complexes were refined using FireDock.^43,44^ Finally, all peptides were docked using HADDOCK 2.4^45^ and the best complexes were selected for a 500 ns molecular dynamics (MD) analysis. HADDOCK 2.4 has several advantages as compared to other docking protocols. HADDOCK score incorporates various energy terms from different stages of the docking protocol, including Rigid Body (it0), Semiflexible Refinement (it1), and Explicit Solvent Refinement (water). Additionally, HADDOCK integrates experimental or bioinformatics-derived restraints, allows flexibility in side chains and backbone regions, and evaluates the quality of the docked complexes. Protein and the best peptide structures are presented in Figure 2. The best complexes are typically identified based on the lowest HADDOCK score, indicating optimal binding interactions between the protein and ligand. For comparison, we utilized HIP000, an experimentally proven HIV-1 integrase inhibitor peptide (FHNHGKQ), as a positive control,^38^ and an experimentally validated negative control peptide, HIP00N (FHNHAKQ),^33,46,47^ was also considered for better comparison.

### Molecular Dynamic (MD) Simulation

A 500 nanosecond (ns) MD simulation was conducted on seven selected integrase protein complexes (IN-HIP000, IN-HIP1113, IN-HIP1140, IN-HIP1142, IN-HIP678, IN-HIP776, and IN-HIP777). Then, two independent simulations were subjected to HIP777, HIP776, HIP1142, and HIP00 complexes for a better understanding of the peptide–protein dynamics properties, as they revealed the best complexes at the time of the initial 500 ns simulation. Initial 500 ns simulations were carried out using the Desmond program,^48^ utilizing the NPT ensemble for system relaxation and a temperature of 300 K and a pressure of 1 bar. The OPLS2005 force field was employed to calculate the system, which was solvated using SPC^49^ water with a 10 Å buffer distance. Neutralization was achieved by adding Na+ and Cl– ions, and to mimic physiological conditions, 0.15 M NaCl was included. Subsequently, for the two independent 1 μs simulations of HIP777, HIP776, HIP1142, and HIP00, the system was solvated with the TIP3P water model at a temperature of 310 K. During the simulation, the M- SHAKE^50^ method was implemented to satisfy hydrogen bonding geometry restrictions, with time steps of 2.0 fs. The long-range electrostatic interactions were computed using the K-space Gaussian split Ewald method^51^ and a van der Waals cutoff of 9 Å, while short-range nonbonded interactions were calculated by the r-REPA integrator,^52^ with a cutoff distance of 9 Å. The long-range and short-range forces were updated every 2 and 6 fs, respectively. Snapshots were taken every 500 ps to analyze the trajectory data and observe the dynamic nature and structural changes of the complexes through the root-mean-square deviation (RMSD), radius of gyration (*R*_g_), root-mean-square fluctuation (RMSF), and solvent-accessible surface area (SASA) metrics.

### MM/GBSA Calculation per Residue

Binding free energy with their residue contribution to this energy was calculated by the web server HawkDock.^53,54^ The final 500 ns snapshot of the peptide complexes was considered for the calculation. The binding free energy (Δ*G*_bind_) was calculated using the MM/GBSA method, which was expressed as follows:
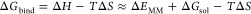
1
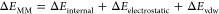
2
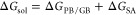
3where Δ*E*_MM_ is the changes of the gas phase MM energy, Δ*G*_sol_ is the solvation free energy, and −*T*Δ*S* the conformational entropy upon binding (eq 1). Δ*E*_MM_ includes Δ*E*_internal_ (bond, angle, and dihedral energies), Δ*E*_electrostatic_ (electrostatic), and Δ*E*_vdw_ (van der Waals) energies (eq 2). Δ*G*_solv_ represents the sum of electrostatic solvation energy (polar contribution), Δ*G*_PB/GB_, and the nonelectrostatic solvation component (nonpolar contribution), Δ*G*_SA_ (eq 3). The polar contribution is calculated using either the GB or PB model, while the nonpolar energy is estimated by SASA.

### Principal Components Analysis

The Principal Components Analysis (PCA) was conducted using a molecular dynamics (MD) trajectory to address the variation in the energy profile and structural fluctuation.^55^ The last 100 ns of the MD trajectory of Coulomb, bond, angles, dihedral, and van-der-Waals energy were considered for the PCA analysis. The outcome of the PCA model can be expressed by the equation.
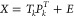
4where *X* represents the combined outcome of multivariate factors, *T_k_* represents the correlation among the samples, *P_k_* is the loading matrix representing the correlation among the variables, *k* is the number of factors included in the model, and *E* represents the unmodeled variance (eq 4). The analysis was performed employing Origin(Pro) version 2023 software packages.

### Structure–Activity Relationship (SAR) Analysis

The SAR is a mathematical model that statistically links the structural features of a compound to its biological properties.^56^ In this study, we identified 30 optimal peptides based on their docking scores for SAR analysis. Various tools such as ProtParam, Peptide Property Calculator, Gravy Calculator, and PepCalc were employed to extract various properties of these peptides, including the distribution of acidic (A), basic (B), aromatic (AR), nonpolar (NP), and polar (P) amino acids, molecular weight, extinction coefficient, approximate volume, and net charge at pH 7.^57−60^ A stepwise multiple linear regression was performed to examine the relationship between the variables and the docking score. Subsequently, the SAR was correlated with the docking score through multiple linear regression analysis.^61,62^ Finally, a principal component analysis biplot was generated based on the four most important properties of the peptides.

## Results and Discussion

### Docking-Derived Peptides Binding Affinity

An initial screening of 280 HIV-1 IN inhibitory peptides was carried out based on their inhibitory concentrations (Table S1). The top 80 peptides were then subjected to further analysis using the HADDOCK 2.4^45^ and they were docked against the HIV-1 IN (PDB ID: 1EXQ). The scores are ranging from −226.5 ± 16.9 to −55 ± 7.7 kcal/mol (Table S2). Among these peptides, HIP1142 (−226.5 ± 16.9), HIP678 (−190.8 ± 16.8), HIP776 (−184.5 ± 28.0), HIP1113 (−184.5 ± 10.1), HIP1140 (−183.0 ± 9.0), and HIP777 (−181.2 ± 10.8) showed maximum HADDOCK scores. The score of the control peptides, HIP000, was −119.0 ± 2.5 and HIP00N, was −119.2 ± 4.1 (Table 1). For further analysis, these six peptides were selected based on their HADDOCK scores and noncovalent interactions (Tables 1 and S3). All selected peptides showed a noticeable interaction with the binding pocket residues as well as catalytic residues. Moreover, divalent Mg^2+^ ions interact with two or more residues in all the peptides except HIP1113. To explore the residue contributions of the selected top three complexes and the positive control peptide BFE, Hawkdock MM/GBSA analysis was performed. The cumulative binding free energy (BFE) values were then listed in Table 1, which provides a comprehensive understanding of their binding affinities compared to the control peptide. The structural properties of the catalytic core domain of the HIV-1 integrase protein and the structures of the top-six peptides along with the control peptides and their corresponding amino acid sequences are illustrated in Figure 2.

**Table 1:** Docking Scores of the Six Selected Peptides That Showed Higher Affinity toward HIV-1 Integrase Protein in HADDOCK

peptide ID	Haddock score	Hawkdock BFE (kcal/mol)
HIP1142	–226.5 ± 16.9	–33.94
HIP678	–190.8 ± 16.8	
HIP776	–184.5 ± 28.0	–25.42
HIP1113	–184.5 ± 10.1	
HIP1140	–183.0 ± 9.0	
HIP777	–181.2 ± 10.8	–55.31
HIP000	–119.0 ± 2.5	–23.44
HIP00N	–119.2 ± 4.1	

### Monitoring Stability of Docked Complexes Using Molecular Dynamics Simulation

**Figure 3:**
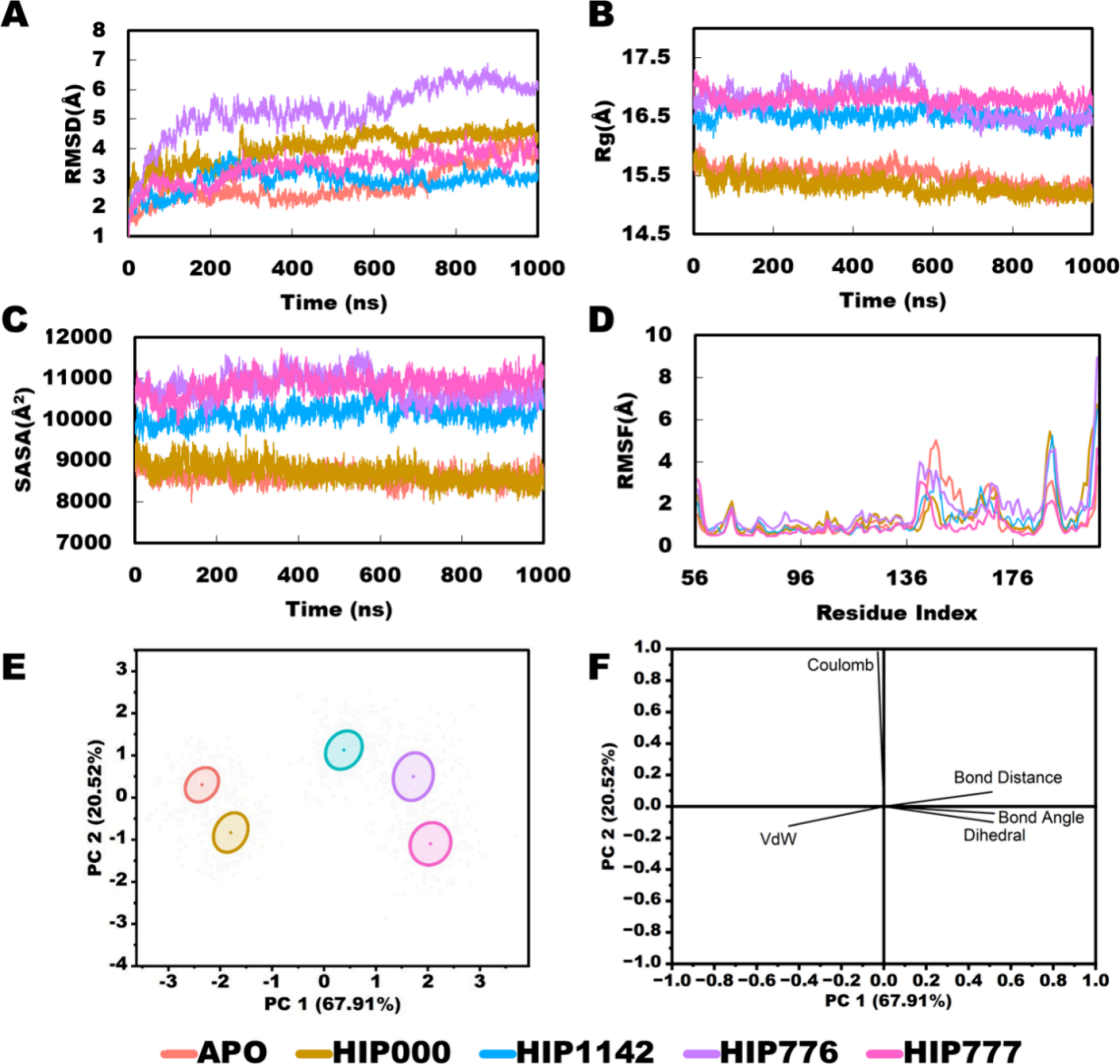
Molecular dynamics simulation of four peptide-integrase complexes with Apo protein. (A) Root mean squared deviation (RMSD) of α carbon; (B) radius of gyration (*R*_g_); (C) solvent accessible surface area (SASA); (D) root mean squared fluctuation (RMSF); (E) PCA scores plot, and (F) loading plot of the energy and structural data from PCA.

A 500 ns molecular dynamics (MD) simulation was implemented on eight IN-peptide complexes (IN-HIP000, IN-HIP00N, IN-HIP1113, IN-HIP1140, IN-HIP1142, IN-HIP678, IN-HIP776, and IN-HIP777) and an apoprotein (IN-apo) to compare the impact of the peptide’s presence on the simulations (Figure S1). Among the eight complexes, HIP777, HIP776, HIP1142, and HIP00 were found stable complexes compared to others. Subsequently, these four complexes, along with the apo protein, underwent multiple 1 μs extended simulations to further investigate their stability and better understand peptide interaction behavior. According to the RMSD profiles, HIP777 and HIP1142 demonstrated the most stable complexes compared to the control peptide HIP00. However, HIP776 exhibited slightly higher RMSD values compared to the other complexes and the apo protein. Interestingly, HIP777 and HIP00 appeared to merge with the apo protein toward the end of the simulation period. Despite HIP1142 initially displaying a slightly higher RMSD, it exhibited the highest stability after 700 ns, even surpassing that of the apo protein (Figure 3A).

In terms of radius of gyration (*R*_g_) and solvent-accessible surface area (SASA) profiles, the complexes can be divided into two groups. The apo protein and HIP00 form one group, demonstrating similar profiles and merging with each other. These complexes maintain lower Rg and SASA profiles compared to the other group (Figure 3B, C). In contrast, HIP777, HIP776, and HIP1142 form the second group, displaying higher *R*_g_ and SASA profiles compared to the control and apo protein. This is attributed to the larger overall complex size of these peptides, containing 20 or more amino acids, whereas the control peptide only consists of 7 amino acids (Figure 3B, C). This observation is further supported by PCA analysis, indicating a distinct clustering behavior (Figure 3E). Interestingly, residue-based root-mean-square-fluctuation (RMSF) values were similar for all complexes, with a consistent fluctuating trend observed in the loop regions across all complexes (Figure 3D).

**Figure 4:**
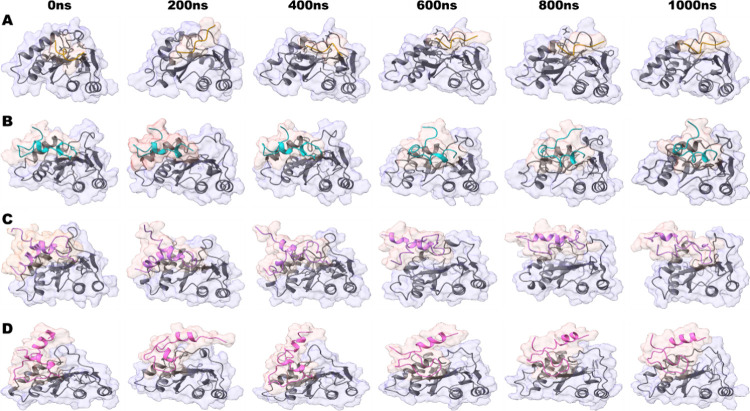
Representative snapshots of four complexes. Snapshots of the simulation were taken in 200 ns intervals starting from 0 to 1000 ns simulation. (A) IN-HIP000 (Citrus); (B) IN-HIP1142 (Deep Sky Blue); (C) IN-HIP776 (Heliotrope); and (D) IN-HIP777 (Neon Pink). Snapshots were taken using ChimeraX.^70^

**Figure 5:**
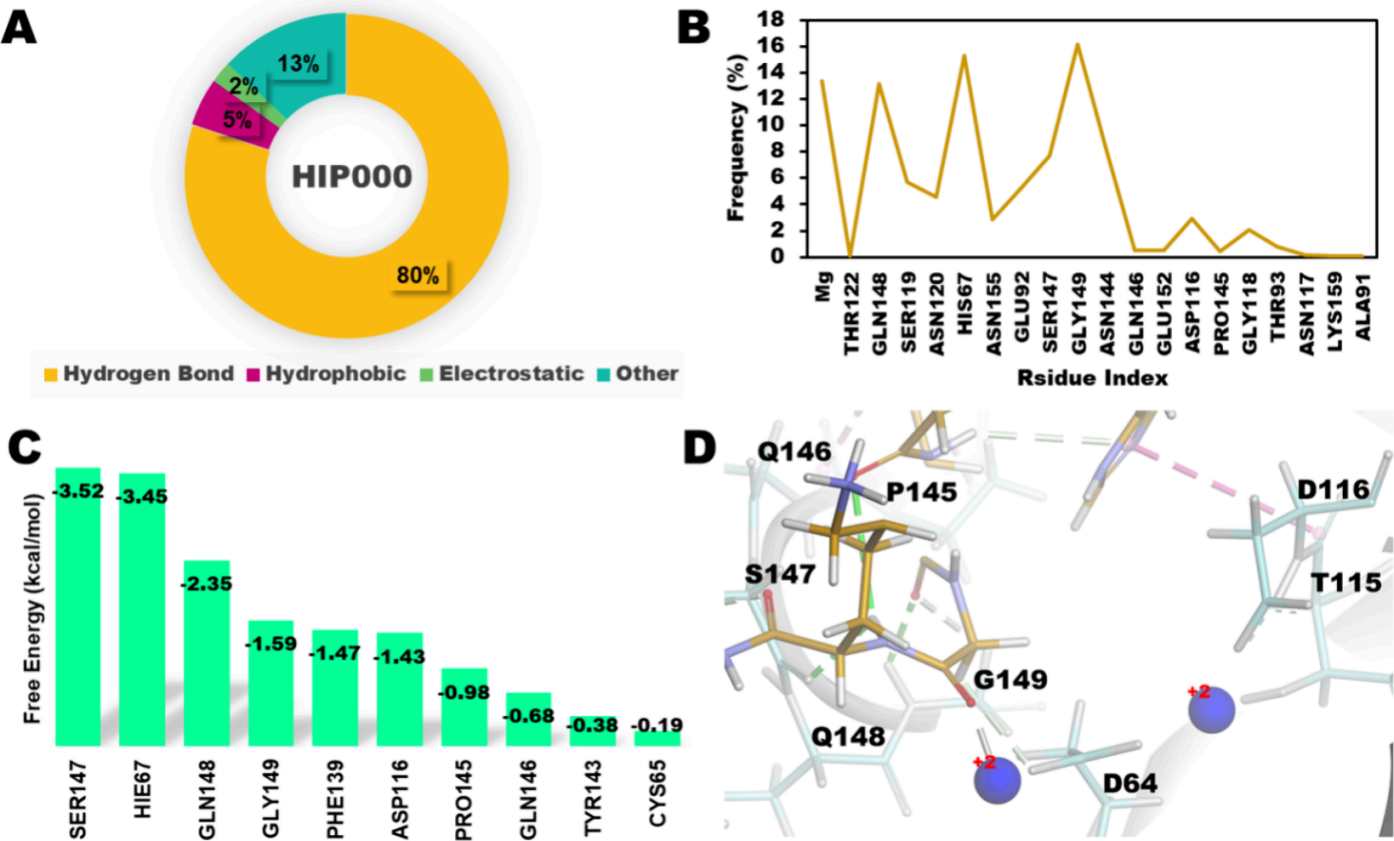
Integrase-HIP000 Complex. (A) Distribution of noncovalent interactions; (B) interacting residues of HIP000; (C) free energy distribution of top ten residues; (D) representative snapshot at 500th ns: IN (gray), HIP000 (Citrus) and Mg^2+^ (blue).

**Figure 6:**
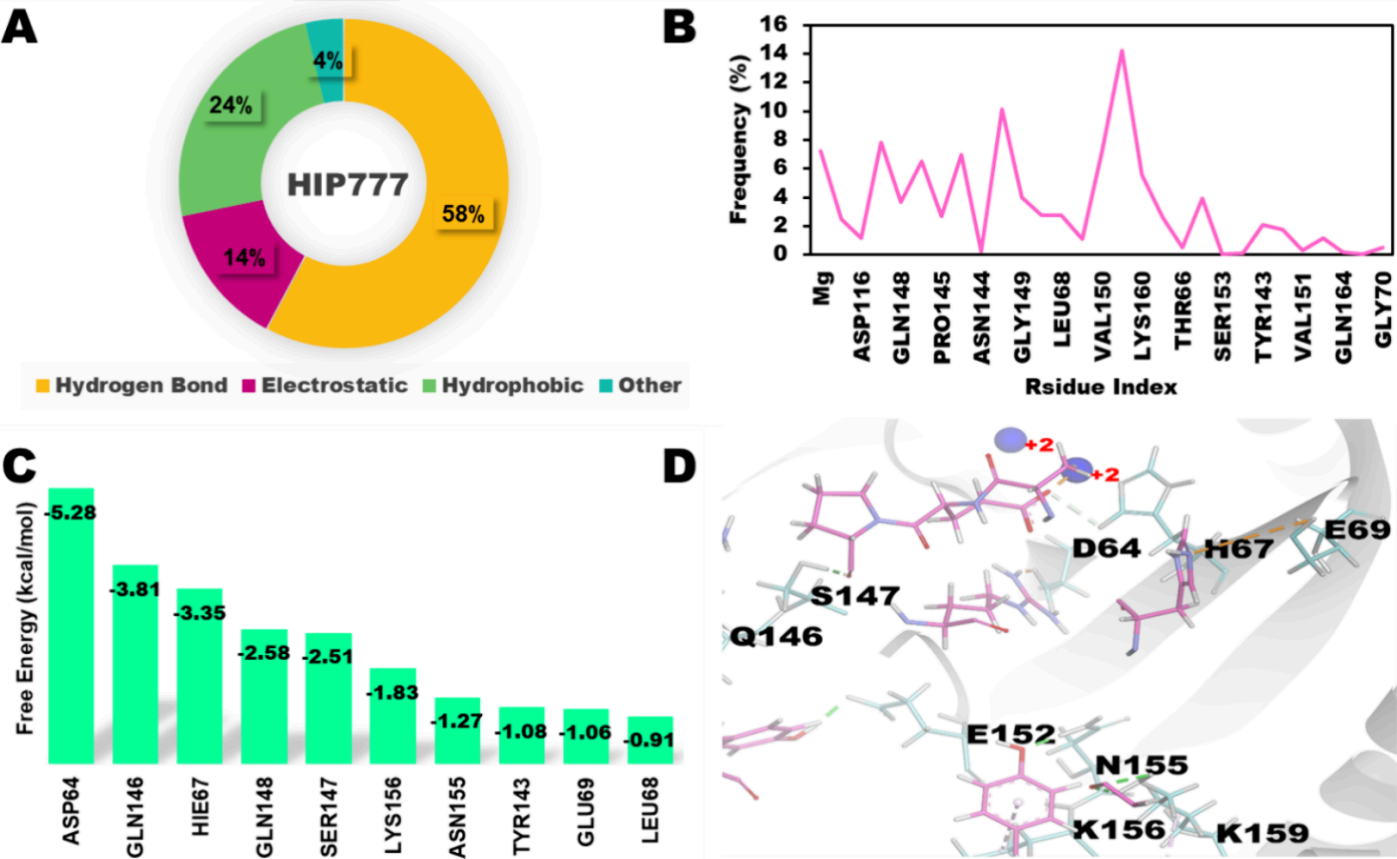
Integrase-HIP777 Complex. (A) Distribution of noncovalent interactions; (B) interacting residues of HIP777; (C) free energy distribution of top ten residues; and (D) representative snapshot at 1000th ns: IN (gray), HIP777 (neon pink), and Mg^2+^ (blue).

**Figure 7:**
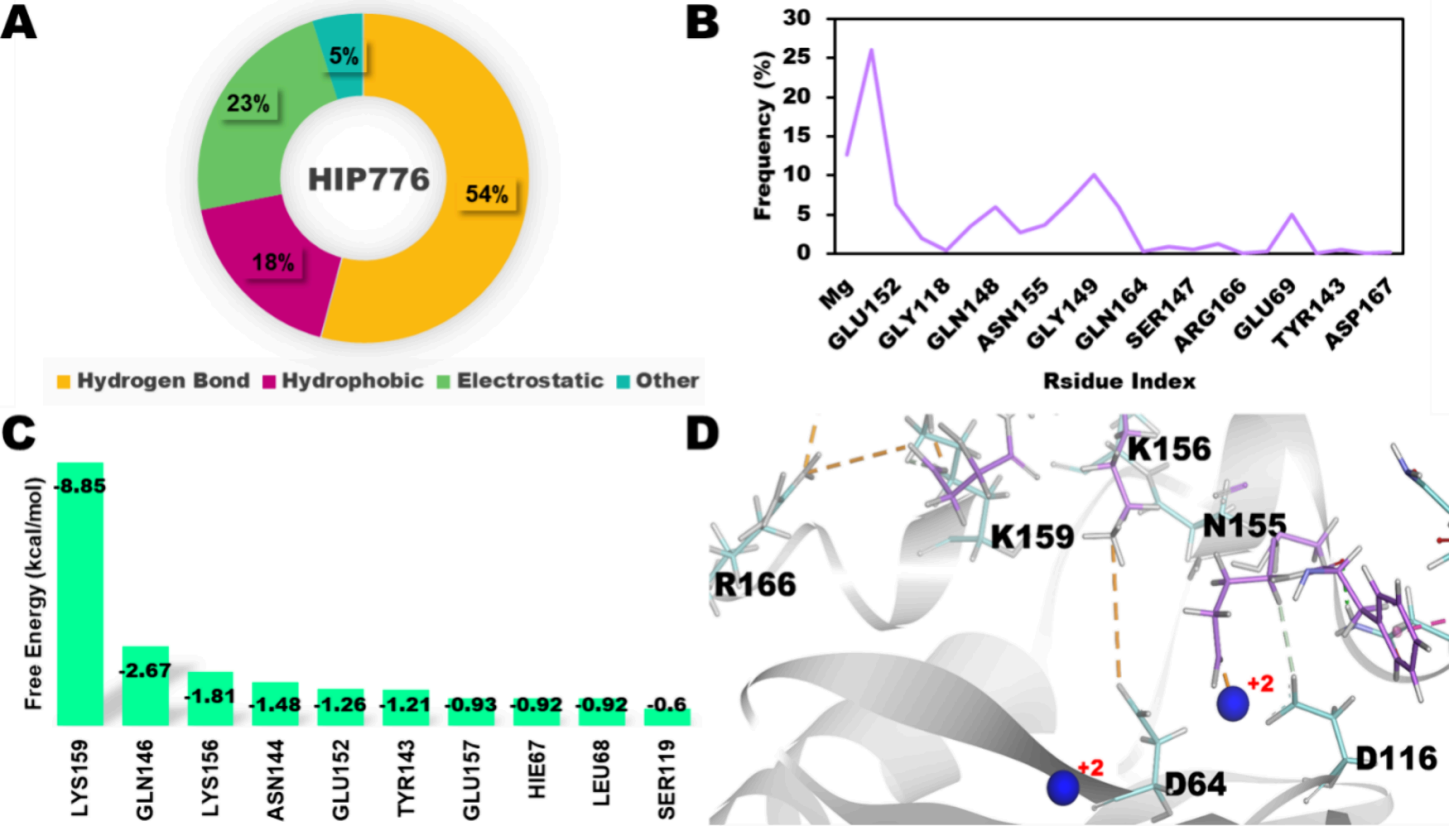
Integrase-HIP776 Complex. (A) Distribution of noncovalent interactions; (B) interacting residues of HIP776; (C) free energy distribution of top ten residues; (D) representative snapshot at 1000th ns: IN (gray), HIP776 (Heliotrope), and Mg^2+^ (blue).

These 3 best-selected complexes and control peptide complexes showed significant interaction with key parameters such as catalytic residues, binding pocket residues, and Mg^2+^ ions and were stable as confirmed by snapshot analysis over the 1 μs simulation time (Figures 5D, 6D, 7D, and S2D). The negative control (HIP00N) was excluded from further analysis as it was shifted from the binding pocket after a few nanoseconds and remained stable there, confirmed by snapshot analysis and the trajectory movie visualization (Figure S6, Movie SM1).

### Residue Interaction Profiles of the Selected Complexes

The 1 μs MD simulation results showed that the IN-HIP777 and IN-HIP776 complexes had superior interaction profiles compared to the other complexes. During the simulation period, several residues in both complexes, including LYS159, LYS156, VAL150, and GLU69, showed strong interactions with the Mg^2+^ ions and catalytic residues of IN (ASP64, ASP116, and GLU152) (Figures 6B, D and 7B, D). Additionally, residues HIS67, GLN46, LYS160, and LYS160 in the IN-HIP777 complex (Figure 6B) and GLN148, GLU152, and ASN155 in the IN-HIP776 complex (Figure 7B), showed interactions with IN throughout the simulation. The IN-HIP777 and HIP000 complexes demonstrated interactions among the three catalytic residues, ASP64, ASP116, and ASP152 (Figures 5B and 6B). In addition, the IN-HIP1142 complex also exhibited strong interaction with LEU68, GLY70, and GLY149 residues and maintained a stable interaction during the simulation period (Figure S2B,D).

The IN-HIP777 complex showed the highest level of interaction in terms of residue interaction analysis, followed by the IN-HIP776, IN-HIP1142, and IN-HIP000 complexes. During the simulation, the hydrogen bonding, hydrophobic, electrostatic, and other bonds in both IN-HIP777 and IN-HIP776 accounted for approximately 55, 25, 15, and 5% of the interactions (Figures 6A and 7A), respectively. Although the IN-HIP1142 complex showed a considerable interaction over the simulation period, it exhibited less than 50% hydrogen bonding (Figure S2A).

### Calculating Binding Free Energy with Key Residue Contribution

The binding free energy and key residue contributions were calculated using the Hawkdock server.^54^ Based on the binding free energy calculation of the last (1 μs) snapshots, the HIP777 peptides showed the highest interaction with the integrase protein, followed by HIP1142, HIP776, and HIP000 peptides. The top 10 residue contribution analysis revealed that the control peptide (HIP000) had the highest number of contributing residues as SER147, HIS67, GLN148, PHE139, and ASP116 (Figure 5C). The HIP777 peptide showed almost similar patterns, with residues ASP64, GLN146, HIS67, GLN148, and SER147 contributing as binding pocket residues (Figure 6C). In the IN-HIP776 complex, the major contributing residue was LYS159 with other minor significant contributions noticed from GLN146, LYS158, ASN144, and GLU152, which are binding pocket residues (Figure 7C). In the HIP1142 peptide, the most contributing residues were HIS67, LYS156, ASN144, VAL150, and ASN120 (Figure S2C). The residue analysis revealed that HIP777, HIP776, and HIP000 peptides showed almost similar interaction profiles with binding pocket residues as well as catalytic residues. Especially, HIP777 showed a higher frequency of interaction with the catalytic residues compared to any other complexes. These insides indicate that the above-mentioned peptides would be promising compared to others, based on the major three parameters (hydrogen bonding, catalytic residues, and Mg^2+^) and representative snapshots (Figures 4 and 9). This residue-based BFE calculation revealed that HIE67, GLN148, GLN146, and SER147 residues contributed most among the selected peptide–protein complexes.

### Principal Component Analysis (PCA)

The principal component analysis (PCA) was performed to investigate the structural and energy profile variations among the peptide–protein complexes compared to the apo-protein. The PCA model was constructed using five data sets, including IN-Apo, three IN-peptide complexes, and the control peptide complex. Over the simulation period, five energy and structural variables namely, Coulomb, bond angles, bond distance, dihedral angles, and Van-der-Waals forces, were included. Those variables were used to generate the score plot of PCA, shown in Figure 3E. The PCA graph revealed that PC1 and PC2 represented 67.91 and 20.52% of the variance, respectively (Figure 3E). The score plot correlated the peptide–protein complexes into two groups: one with the control peptide and apo-protein (APO and HIP000), and another with the rest of the three peptides (HIP777, HIP776, HIP1142). This clustering pattern is also supported by the *R*_g_ and SASA profiles of the complexes (Figure 3B, C). Staying in a similar group of best-selected peptides indicates that they have similar structural and energy profiles (Figure 3E). The loading plot of PCA, shown in the figure, revealed that van der Waals negatively correlates with PC1, whereas Coulomb positively correlates with the PC2. These two energy profiles of the peptide complexes may be the key reason for the shifting (Figure 3F).

### Structure–Activity Relationship (SAR)

**Figure 8:**
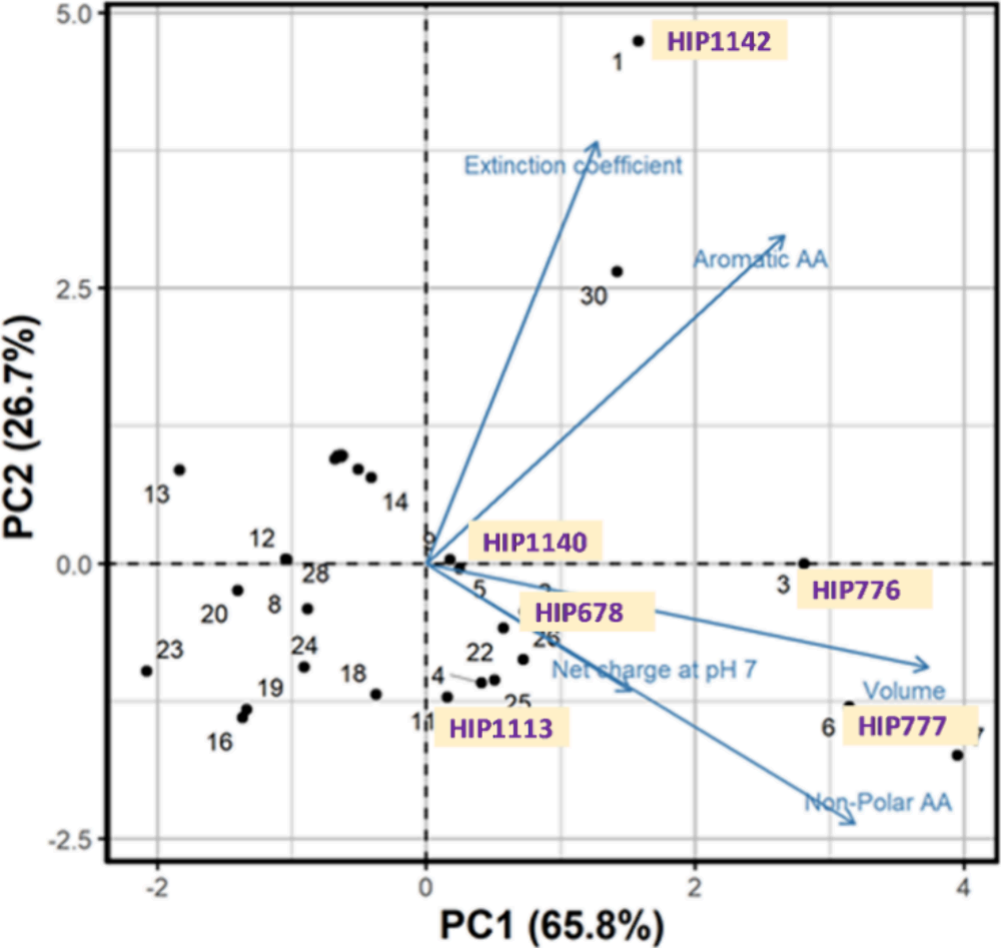
Biplot of the selected 30 high binding affinity peptides clustered based on four peptide properties.

A multiple linear regression (MLR) analysis was implemented to determine the significant predictors of the binding affinity of test peptides based on the most relevant peptide features. This popular bioinformatics technique for predictive pattern analysis^63,64^ was utilized to uncover the pattern of potential peptide ligands (Table S4). Additionally, the principal component analysis (PCA) was performed based on the five principal properties of the peptides that the MLR suggested. The volume, net charge at pH 7, aromatic amino acid, extinction coefficient, and nonpolar amino acid were identified as the most significant predictors of the protein-peptide complexes and represented the dominant characteristics of hydrogen, hydrophobic, and electrostatic interactions. The aromatic amino acids and overall volume of peptides were found to have a positive correlation with the HADDOCK scores, suggesting that an increase in the aromatic residues and volume of peptides leads to enhanced substrate interactions, which is supported by several studies.^65−68^ Conversely, the net charge at pH 7 and extinction coefficient negatively correlated with the HADDOCK scores, implying that an increase in these properties weakens the interactions. The volume, nonpolar amino acid, and net charge at pH 7 heavily loaded in the first principal component, which accounted for approximately 66% of the variability. The second component was dominated by the extinction coefficient and aromatic amino acids, accounting for about 27% of the variability. The PCA score plot revealed that complexes with similar peptide properties, such as HIP1140, HIP1113, and HIP678, formed a cluster, while HIP776 and HIP777 formed another cluster (Figure 8). This supports the RMSD and Rg profiles of these complexes (Figure 3). However, HIP1142 showed deviation in the energy score plot and was distinct from the rest of the complexes. Overall, volume and aromatic amino acids may play a significant role in protein–ligand interactions, as the most stable complexes, HIP777 and HIP776, cluster in areas dominated by these two properties.

**Figure 9:**
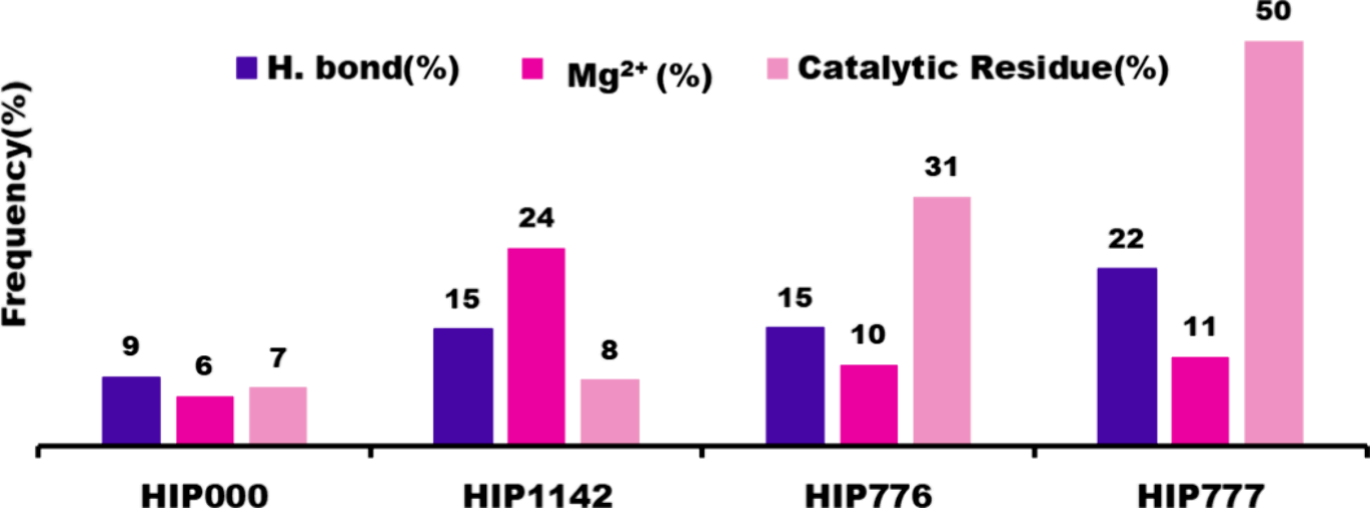
Three important parameters (hydrogen bond, Mg^2+^, and catalytic residues) comparison among the seven complexes over 500 ns simulation.

### Peptide Conformational Variation

#### Peptide HIP000

**Figure 10:**
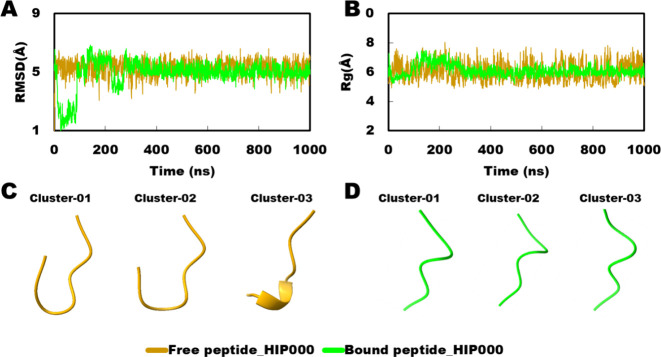
Comparison of HIP000 peptide conformation on free and bound state. (A) RMSD in the free and bound state; (B) *R*_g_ in free and bound state; (C) best three trajectory clusters in the free state; and (D) best three trajectory clusters in the bound state.

For the positive control peptide, the RMSD and *R*_g_ graphs showed merging between the free and bound states, suggesting an overall similarity in the peptide’s conformation (Figure 10A, B). However, a significant difference was observed in the conformation between the free and bound states. This discrepancy could be attributed to the influence of protein-peptide interactions, which stabilize the peptide’s conformation in the bound state. Upon examining the best three clusters in each state, it was evident that in the bound state, the peptide conformers were similar, reflecting the stabilizing effect of protein binding. In contrast, in the free state, the three clusters exhibited distinct conformations, each differing from the native state, indicating the peptide’s ability to explore different conformations in the absence of protein interaction (Figure 10C, D).

#### Peptide HIP777

**Figure 11:**
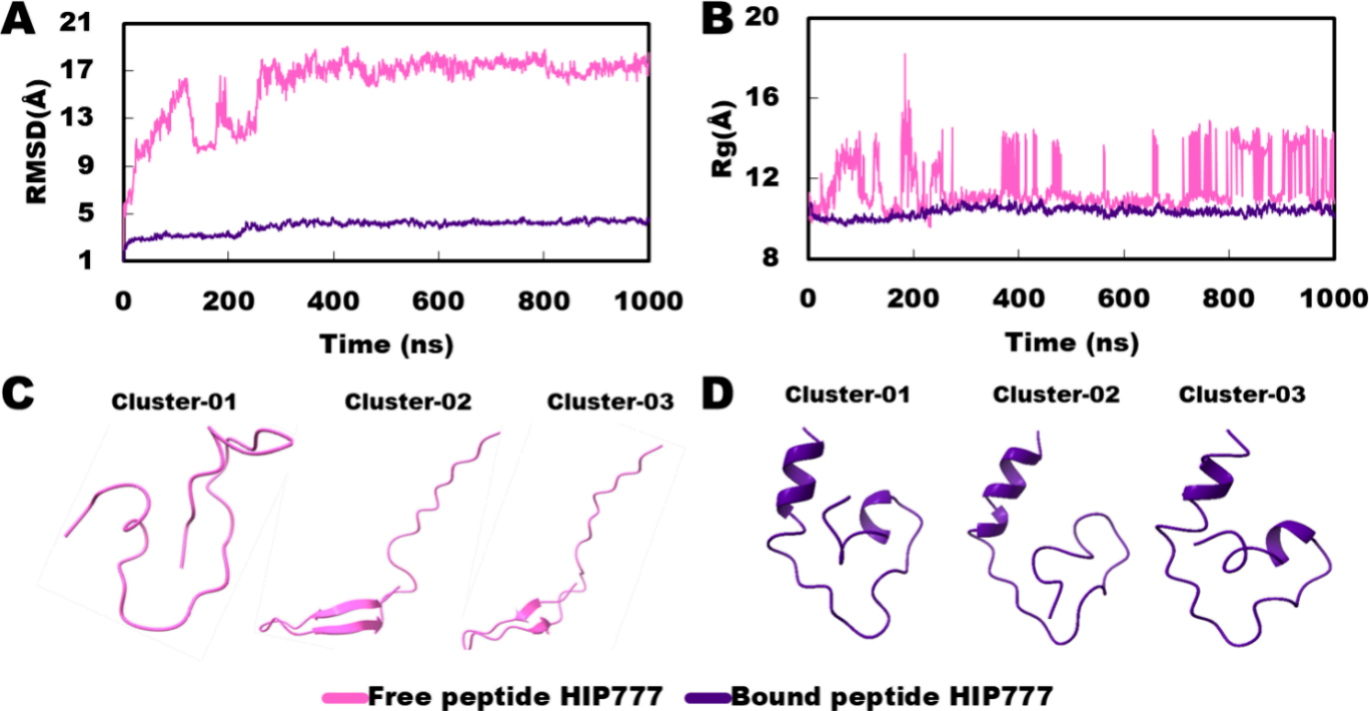
Comparison of HIP777 peptide conformation on free and bound state. (A) RMSD in the free and bound state; (B) *R*_g_ in the free and bound state; (C) best three trajectory clusters in the free state; and (D) best three trajectory clusters in the bound state.

For HIP777, which is quite similar to the positive control peptide conformation changes although there are significant differences and fluctuations observed in both RMSD and Rg between the free and bound states (Figure 11A, B). In the bound state, the peptide conformers across the three clusters closely resembled the native state, indicating stabilization of the conformation by interactions with the protein. However, in the absence of protein binding, the peptide underwent significant conformational changes divergent from the native state. This discrepancy underscores the influence of protein–peptide interactions on the peptide’s conformational dynamics. The stability of the peptide’s conformation in the bound state suggests strong interactions that restrict conformational changes, emphasizing the crucial role of protein–peptide interactions in maintaining structural integrity (Figure 11C, D).

#### Peptide HIP776

**Figure 12:**
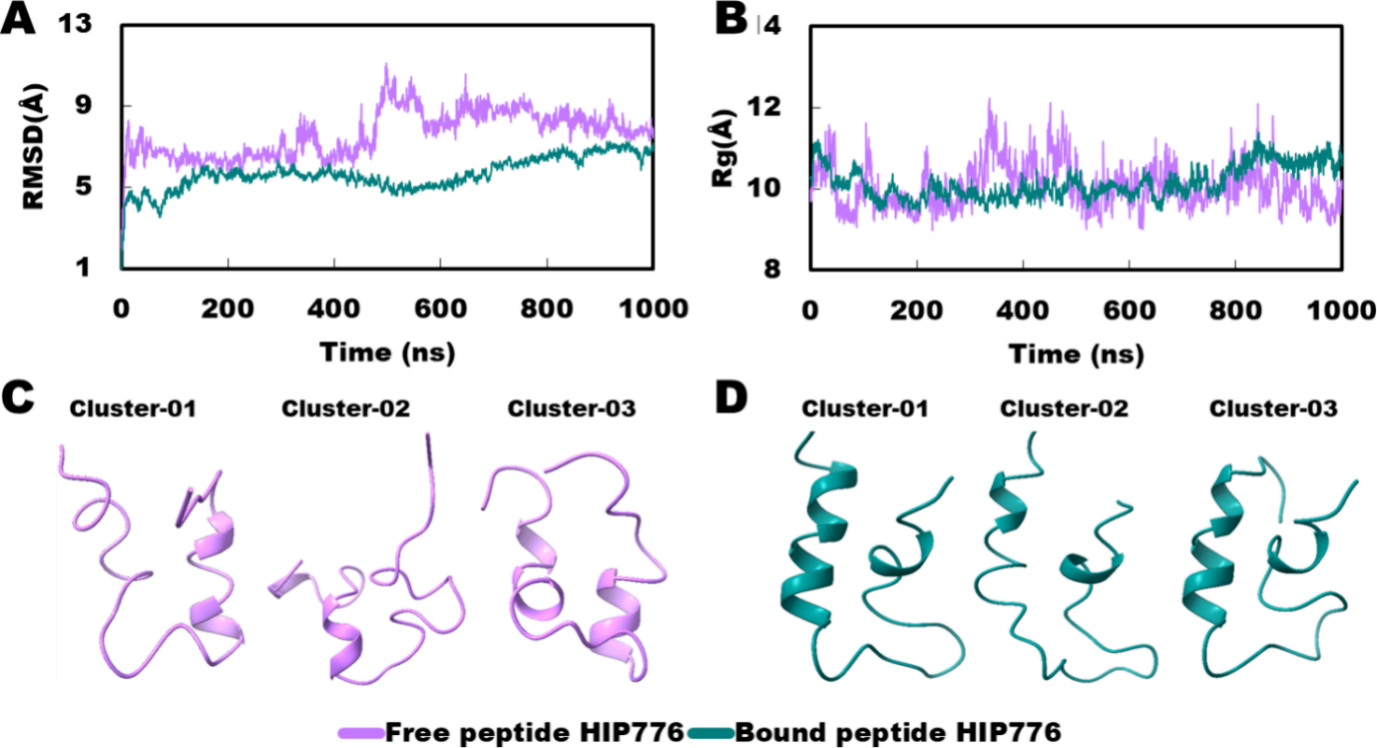
Comparison of HIP776 peptide conformation on free and bound state. (A) RMSD in the free and bound state; (B) *R*_g_ in the free and bound state; (C) best three trajectory clusters in free state; and (D) best three trajectory clusters in bound state.

In the case of HIP776, the RMSD graph displayed a merging of a few points with the final native state, indicating some similarity between the free and bound states. Similarly, the *R*_g_ graph showed almost complete merging between the free and bound states (Figure 12A, B). Furthermore, in the bound state, the conformers of the peptide across the three clusters closely resembled the native state, indicating stable protein-peptide interactions. Surprisingly, in the free state, the clusters exhibited conformations that were also quite similar to those observed in the bound state, suggesting that the peptide may have inherent structural stability even in the absence of protein binding.

#### Peptide HIP1142

In the case of HIP1142, the RMSD graphs show differences between the free and bound states, yet both exhibit similar stable patterns without significant fluctuations within each graph (Figure S4A). Additionally, the Rg graphs demonstrate merging between the free and bound states, indicating similarity in the overall peptide size across both states, which is also observed in cluster visualization (Figure S4B). All clusters of HIP1142 exhibit nearly identical conformations in both the free and bound states, suggesting consistent stability regardless of its binding status to the protein. These results imply that HIP1142 maintains stability and uniformity in conformation between the free and bound states, with minimal conformational alterations observed upon binding to the protein, indicating the presence of a relatively rigid structure in HIP1142 that persists regardless of its binding status (Figure S4C,D).

## Conclusions

This study utilized a computational approach to identify peptide mechanisms and a few peptides that could be utilized for future structure-based drug design against the integrase of HIV. Through molecular docking and molecular dynamics simulations, six peptides, namely HIP1142, HIP678, HIP776, HIP1113, HIP1140, and HIP777, were identified as top candidates based on their favorable binding affinities and stable interactions with the target protein. The best three HIP777, HIP776, and HIP1142 utilized for an in-depth mechanism study through multiple 1 μs simulations. The simulations revealed significant differences in conformational dynamics between the free and bound states of these selected three peptides, highlighting the impact of protein-peptide interactions on structural stability. HIP777 peptide exhibited a stabilizing effect on conformation in the bound state, while HIP776 demonstrated inherent structural stability even in the absence of protein binding. Residue interaction analysis and binding free energy calculations elucidated the key residues involved in peptide binding and highlighted the significance of specific interactions in stabilizing the complexes. Structure–activity relationship analysis revealed the importance of aromatic amino acids and peptide volume in determining binding affinities. Overall, these findings provide valuable insights into the molecular mechanisms underlying peptide–protein interactions and pave the way for the development of novel antiviral therapeutics targeting HIV-1 integrase. Experimental structural validation will be crucial for further peptide mechanism exploration, addressing the urgent need for effective HIV treatments.

## Supporting Information Available

Detailed data and analyses; sequence, length, and potency of 280 integrase inhibitor peptides against various HIV-infected cell lines; binding scores of 80 peptides against the HIV-1 integrase protein; details of the nonbonding interactions of the best six docking complexes with a control peptide; results of a stepwise multiple linear regression analysis, identifying the most relevant peptide properties as predictors of binding affinity; visual data from molecular dynamics simulations, including RMSD, radius of gyration, solvent accessible surface area, and noncovalent interactions, along with a trajectory movie (SM1) illustrating the deviation of a negative control peptide from the binding pocket; HIP776 peptide’s conformation in free and bound states; and simulation analysis variables from two independent runs (PDF)
Trajectory movie (SM1) illustrating the deviation of a negative control peptide from the binding pocket (MP4)

## Accession Codes

During the preparation of this work the author(s) used OpenAI ChatGPT to improve readability of the manuscript. After using this tool/service, the author(s) reviewed and edited the content as needed and take(s) full responsibility for the content of the publication.
