# Supplementary material for: Structure-Guided Antiviral Peptides Identification Targeting the HIV-1 Integrase
Source: ACS Phys Chem Au. 2024 Jul 5;4(5):464–75. doi: 10.1021/acsphyschemau.4c00006 (PMC11428276; doi:10.1021/acsphyschemau.4c00006)
Supplement: Supplementary file 1 — pg4c00006_si_001.pdf [file pg4c00006_si_001.pdf]

Structure-Guided Antiviral Peptides Identification Targeting the HIV-1 Integrase

Md. Shahadat Hossain[a,b§], Md. Siddik Alom[c,d§], Mohammad Salauddin Kader[e], Mohammed Akhter Hossain[f], Mohammad A. Halim[g*]

[a]Division of Infectious Diseases and Division of Computer Aided Drug Design, The Red-Green Research Centre, BICCB, 16 Tejkunipara, Tejgaon, Dhaka 1215, Bangladesh.
[b]Department of Pharmacy, Faculty of Life Science, Mawlana Bhashani Science & Technology University, Tangail 1902, Bangladesh.
[c]Ohio State Biochemistry Program, The Ohio State University, Columbus, Ohio 43210, USA.
[d]Center for RNA Biology, The Ohio State University, Columbus, Ohio 43210, USA.
[e]Department of Chemistry, Saint Louis University, Saint Louis, Missouri 63101, USA.
[f]The Florey, University of Melbourne, Melbourne, Victoria 3010, Australia.
[g]Department of Chemistry and Biochemistry, Kennesaw State University, Kennesaw, Georgia 30144, USA.

[§]*M.S.H. and M.S.A. contributed equally to this paper.*

* Corresponding Author: mhalim1@kennesaw.edu

Table S1. Sequence, length, potency of the 280 integrase inhibitor peptides against various cell lines infected with HIV virus (retrieved from HIPdb).

HIV Integrase inhibitory peptides.

Peptide ID	Sequence	Length	Cell line	Inhibition/IC50	Unit
HIP43	HCKFWD	6	E.coli	18	%
HIP46	HCKFWE	6	E.coli	28	%
HIP61	HCKFWN	6	E.coli	30	%
HIP41	HCKFWC	6	E.coli	30	%
HIP34	HAKFWW	6	E.coli	30	%
HIP69	HCKFWS	6	E.coli	40	%
HIP60	HCKFWM	6	E.coli	42	%
HIP64	HCKFWP	6	E.coli	45	%
HIP52	HCKFWH	6	E.coli	72	%
HIP78	HCKFWY	6	E.coli	75	%
HIP50	HCKFWG	6	E.coli	82	%
HIP73	HCKFWV	6	E.coli	88	%
HIP68	HCKFWR	6	E.coli	95	%
HIP48	HCKFWF	6	E.coli	96	%
HIP54	HCKFWI	6	E.coli	96	%
HIP1049	SLKIPNLD	8	NA	18	%
HIP44	HCKFWD	6	NA	24	%
HIP45	HCKFWE	6	NA	30	%
HIP42	HCKFWC	6	NA	30	%
HIP55	HCKFWK	6	NA	30	%
HIP62	HCKFWN	6	NA	40	%
HIP59	HCKFWM	6	NA	40	%
HIP70	HCKFWS	6	NA	40	%
HIP63	HCKFWP	6	NA	42	%
HIP51	HCKFWH	6	NA	69	%
HIP79	HCKFWY	6	NA	78	%
HIP49	HCKFWG	6	NA	79	%
HIP58	HCKFWL	6	NA	83	%
HIP74	HCKFWV	6	NA	85	%
HIP47	HCKFWF	6	NA	92	%
HIP53	HCKFWI	6	NA	92	%
HIP77	HCKFWW	6	NA	100	%

HIP1018	WQCLTLTHRG	10	HeLa	68	%
HIP1017	WQCLTLTHRGFVLLTITVLR	20	HeLa	70	%
HIP1031	WNSLKIANLAV	11	Sup-T1	43	%
HIP1027	WNSLKIANLDV	11	Sup-T1	58	%
HIP1026	WNSLKADNLDV	11	Sup-T1	65	%
HIP1030	WNSLKIDNLDA	11	Sup-T1	68	%
HIP1025	WNSAKIDNLDV	11	Sup-T1	71	%
HIP1029	WNSLKIDNADV	11	Sup-T1	75	%
HIP1028	WNSLKIDALDV	11	Sup-T1	75	%
HIP1023	WASLKIDNLDV	11	Sup-T1	76	%
HIP1024	WNALKIDNLDV	11	Sup-T1	79	%
HIP1022	WNSLKIDNLDV	11	Sup-T1	81	%
HIP104	STPIPAP	7	NA	>10^12	FC50(pfu/mL)
HIP113	FHNHGAP	7	NA	>10^12	FC50(pfu/mL)
HIP117	FHNHGST	7	NA	>10^12	FC50(pfu/mL)
HIP99	RLFTWEG	7	NA	>10^12	FC50(pfu/mL)
HIP92	HIEHLIA	7	NA	10^10	FC50(pfu/mL)
HIP93	HLEHLLF	7	NA	10^10	FC50(pfu/mL)
HIP116	FHNHGKQ	7	NA	10^11	FC50(pfu/mL)
HIP102	SSLPLRK	7	NA	10^12	FC50(pfu/mL)
HIP103	STFTHPR	7	NA	5*10^11	FC50(pfu/mL)
HIP112	FHNHGAA	7	NA	5*10^11	FC50(pfu/mL)
HIP114	FHNHGAT	7	NA	5*10^11	FC50(pfu/mL)
HIP115	FHNHGIL	7	NA	5*10^11	FC50(pfu/mL)
HIP97	PFFHLIG	7	NA	5*10^11	FC50(pfu/mL)

HIP111	FHGHGLY	7	NA	7*10^11	FC50(pfu/mL)
HIP1043	STTVKAASWWA	11	NA	>1	mM
HIP1042	GYIEAEVI	8	NA	>1	mM
HIP1051	WIHAEIKNSLKIDNLDVNRCIEALD	25	NA	High	NA
HIP1056	AGVEAIIRILQQLLF	15	MT-4	High	NA
HIP1057	IIRILQQLLFIHFRI	15	MT-4	High	NA
HIP1058	LQQLLFIHFRIGCQH	16	MT-4	High	NA
HIP1045	SQGVVESMNKELKKIIGQVRDQAEHLKTAY	30	NA	High	NA
HIP1052	MAGRSGDSDEELLKTVRLIKFLYQSNPPPS	30	TZM-bl	High	NA
HIP1053	LKTVRLIKFLY	11	TZM-bl	High	NA
HIP1054	QRQIRSISGWILSTYLGRPAEPVPLQ	26	TZM-bl	High	NA
HIP1055	RSISGWILSTYLGRP	15	TZM-bl	High	NA
HIP66	HCKFWQ	6	E.coli	Low	NA
HIP57	HCKFWL	6	E.coli	Low	NA
HIP72	HCKFWT	6	E.coli	High	NA
HIP39	HCKFWA	6	NA	Low	NA
HIP65	HCKFWQ	6	NA	Low	NA
HIP67	HCKFWR	6	NA	Low	NA
HIP71	HCKFWT	6	NA	Low	NA
HIP620	KILEPFRKQNPDIVIYQYMD	20	NA	4.8	NA
HIP581	ELVNQIIEQLIKKEKVYLAW	20	NA	6.9	NA
HIP700	SPAIFQSSMTKILEPFRKQN	20	NA	35	NA
HIP293	FRKQNPDIVIYQYMD	15	NA	119	NA
HIP610	IQAQPDQSESELVNQIIEQL	20	NA	>120	NA
HIP552	IKKEKVYLAWVPAHKGIGN	19	NA	>120	NA
HIP621	KILEPFRKQNPDIVIYQYMD	20	NA	High	NA
HIP679	PDIVIYQYMDDLYVGSDLEI	20	NA	High	NA
HIP609	IAEIQKQGQGQWTYQIYQEP	20	NA	High	NA
HIP624	KQLTEAVQKITTESIVIWGK	20	NA	High	NA
HIP714	TPKFKLPIQKETWETWWTEY	20	NA	High	NA
HIP727	VVTLTDTTNQKTELQAIYLA	20	NA	High	NA
HIP582	ELVNQIIEQLIKKEKVYLAW	20	NA	High	NA

HIP583	EQVDKLVSAGIRKVLFLDGI	20	NA	High	NA
HIP568	CTLNFPISPIETVPVKLKPG	20	NA	Low	NA
HIP586	ETVPVKLKPGMDGPKVKQWP	20	NA	Low	NA
HIP672	MDGPKVKQWPLTEEKIKALV	20	NA	Low	NA
HIP671	LTEEKIKALVEICTEMEKEG	20	NA	Low	NA
HIP579	EICTEMEKEGKISKIGPENP	20	NA	Low	NA
HIP622	KISKIGPENPYNTPVFAIKK	20	NA	Low	NA
HIP732	YNTPVFAIKKKDSTKWRKLV	20	NA	Low	NA
HIP615	KDSTKWRKLVDFRELNKRTQ	20	NA	Low	NA
HIP571	DFWEVQLGIPHPAGLKKKKS	20	NA	Low	NA
HIP606	HPAGLKKKKSVTVLDVGDAY	20	NA	Low	NA
HIP726	VTVLDVGDAYFSVPLDEDFR	20	NA	Low	NA
HIP590	FSVPLDEDFRKYTAFTIPSI	20	NA	Low	NA
HIP629	KYTAFTIPSINNETPGIRYQ	20	NA	Low	NA
HIP674	NNETPGIRYQYNVLPQGWKG	20	NA	Low	NA
HIP733	YNVLPQGWKGSPAIFQSSMT	20	NA	Low	NA
HIP576	DLYVGSDLEIGQHRTKIEEL	20	NA	Low	NA
HIP601	GQHRTKIEELRQHLLRWGLT	20	NA	Low	NA
HIP697	RQHLLRWGLTTPDKKHQKEP	20	NA	Low	NA
HIP713	TPDKKHQKEPPFLWMGYELH	20	NA	Low	NA
HIP682	PFLWMGYELHPDKWTVQPIV	20	NA	Low	NA
HIP680	PDKWTVQPIVLPEKDSWTVN	20	NA	Low	NA
HIP666	LPEKDSWTVNDIQKLVGKLN	20	NA	Low	NA
HIP573	DIQKLVGKLNWASQIYPGIK	20	NA	Low	NA
HIP722	VRQLCKLLRGTKALTEVIPL	20	NA	Low	NA
HIP710	TKALTEVIPLTEEAELELAE	20	NA	Low	NA
HIP706	TEEAELELAENREILKEPVH	20	NA	Low	NA
HIP675	NREILKEPVHGVYYDPSKDL	20	NA	Low	NA
HIP604	GVYYDPSKDLIAEIQKQGQG	20	NA	Low	NA
HIP690	QWTYQIYQEPFKNLKTGKYA	20	NA	Low	NA
HIP695	RMRGAHTNDVKQLTEAVQKI	20	NA	Low	NA
HIP715	TTESIVIWGKTPKFKLPIQK	20	NA	Low	NA
HIP587	ETWETWWTEYWQATWIPEWE	20	NA	Low	NA
HIP731	WYQLEKEPIVGAETFYVDGA	20	NA	Low	NA
HIP591	GAETFYVDGAANRETKLGKA	20	NA	Low	NA
HIP561	ANRETKLGKAGYVTNRGRQK	20	NA	Low	NA
HIP627	KTELQAIYLALQDSGLEVNI	20	NA	Low	NA
HIP667	LQDSGLEVNIVTDSQYALGI	20	NA	Low	NA
HIP725	VTDSQYALGIIQAQPDQSES	20	NA	Low	NA

HIP718	VPAHKGIGGNEQVDKLVSAG	20	NA	Low	NA
HIP612	IRKVLFLDGIDKAQDEHEKY	20	NA	Low	NA
HIP318	KGSPAIFQSSMTKIL	15	NA	Low	NA
HIP274	AIFQSSMTKILEPFR	15	NA	Low	NA
HIP359	SSMTKILEPFRKQNP	15	NA	Low	NA
HIP319	KILEPFRKQNPDIVI	15	NA	Low	NA
HIP336	PFRKQNPDIVIYQYM	15	NA	Low	NA
HIP343	QNPDIVIYQYMDDLY	15	NA	Low	NA
HIP311	IVIYQYMDDLYVGSD	15	NA	Low	NA
HIP347	QYMDDLYVGSDLEIG	15	NA	Low	NA
HIP306	IQAQPDKSESELVSQ	15	NA	Low	NA
HIP334	PDKSESELVSQIIEQ	15	NA	Low	NA
HIP373	VSQIIEQLIKKEKVY	15	NA	Low	NA
HIP302	IEQLIKKEKVYLAWV	15	NA	Low	NA
HIP304	IKKEKVYLAWVPAHK	15	NA	Low	NA
HIP322	KVYLAWVPAHKGIGG	15	NA	Low	NA
HIP267	AWVPAHKGIGGNEQ	14	NA	Low	NA
HIP294	FRKQNPDIVIYQYMD	15	NA	Low	NA
HIP771	SQGVVESMNKELKKIIGQVRDQAEHLKTA	29	BL21	High	NA
HIP1046	VESMNEELKKIIAQVRAQAEHLKTAY	27	NA	High	NA
HIP1102	RQIKIWFQNRRMKWKK	16	TZM-bl	High	NA
HIP971	VESMNKELPKIIGQVRDQAEHLKTAY	26	NA	High	NA
HIP969	SQGVVESMNKELKKIIGQVRDQAEHLKTAY	30	NA	High	NA
HIP970	SQGVVESMNKELPKIIGQVRDQAEHLKTAY	30	NA	Low	NA
HIP1044	ATGQETAYFLLKLALKA	18	NA	High	NA
HIP1110	LKTVRLIKFLY	11	NA	High	NA
HIP1111	RSISGWILSTYLGRP	15	NA	High	NA
HIP1047	WIHAEIKNSLKIDNLDVNRCIEALD	25	HeLa	High	NA
HIP1048	WKKIRRFVSQVIM	13	HeLa	High	NA
HIP1050	WNSLKIDNLDV	11	NA	High	NA
HIP100	SLLSSPQ	7	NA	Low	NA
HIP101	SPYHTQP	7	NA	Low	NA
HIP105	TTYSRFP	7	NA	Low	NA

HIP107	VPTGYKP	7	NA	Low	NA
HIP109	AEPVAML	7	NA	Low	NA
HIP110	ASSRTPS	7	NA	Low	NA
HIP118	FHQNWPS	7	NA	Low	NA
HIP83	NPRLYE	6	NA	Low	NA
HIP90	HAWNYIF	7	NA	Low	NA
HIP91	HFWNRPL	7	NA	Low	NA
HIP94	HWGMWSY	7	NA	Low	NA
HIP95	LPPNPTN	7	NA	Low	NA
HIP96	NSHAIYP	7	NA	Low	NA
HIP1114	WIHAEIKNSLKIDNLDVNRCIEALD	25	NA	2020	nM
HIP1115	WNSLKIDNLDV	11	NA	11900	nM
HIP1128	LQQLLF	6	MT-4	68?	nM
HIP1113	DQAEHLKTAVQMAVFIHNYKA	21	NA	85	nM
HIP1135	AGERIVDIIATDIQ	14	NA	2	μM
HIP1134	HLKTAVQMAVFIHNFKR	17	NA	3	μM
HIP1133	QETAYFLLKLAGRWP	15	NA	3.5	μM
HIP1137	AGERIVDIIA	10	NA	30	μM
HIP1136	QETAYFLLKLAGR	13	NA	150	μM
HIP1140	PDIVIYQYMDDLYVGSDLEI	21	NA	6	μM
HIP1142	ETWETWWTEYWQATWIPEWE	20	NA	6	μM
HIP1141	KQLTEAVQKITTESIVIWGK	20	NA	7	μM
HIP1143	LQDSGLEVNIVTDSQYALGI	20	NA	11	μM
HIP1144	ELVNQIIEQLIKKEKVYLAW	20	NA	15	μM
HIP75	HCKFWW	6	E.coli	2	μM
HIP36	HCKAWW	6	E.coli	8	μM
HIP56	HCKFWK	6	E.coli	34	μM
HIP35	HCAFWW	6	E.coli	49	μM
HIP30	ACKFWW	6	E.coli	51	μM
HIP37	HCKFAW	6	E.coli	150	μM
HIP38	HCKFWA	6	E.coli	210	μM

HIP678	PDIVIYQYMDDLYVGSDLEI	20	NA	22	μM
HIP288	ESELVSQIIEQLIKK	15	NA	>120	μM
HIP271	NQIIEQLIKKEKVY	14	NA	>240	μM
HIP1138	ATGQETAYFLLKLAGKA	17	NA	250	μM
HIP1059	ILPWKWPWWPWRR	14	NA	60	μM
HIP1060	ILPWKWPWWPWPP	13	NA	60	μM
HIP88	YFLLKL	6	NA	20	μM
HIP84	TAYFLL	6	NA	500	μM
HIP404	HGQVDCSPGIWQLDCTH	17	NA	1000	μM
HIP81	KLAGRW	6	NA	>100	μM
HIP31	ACWWAG	6	NA	>100	μM
HIP87	WAGIKQ	6	NA	>100	μM
HIP26	IKQEF	5	NA	>100	μM
HIP170	ACWWAGIKQEF	11	NA	>1000	μM
HIP152	FGIPYNPQSQ	10	NA	>1000	μM
HIP156	WKGPAKLLWK	10	NA	>1000	μM
HIP150	EEHEKYHSNW	10	NA	>2000	μM
HIP151	ESMNKELKKI	10	NA	>2000	μM
HIP155	VRDQAEHLKT	10	NA	>2000	μM
HIP122	FIHNFKRK	8	NA	>2000	μM
HIP153	GYSAGERIVD	10	NA	>2000	μM
HIP149	ASCDKCQLKG	10	NA	>2000	μM
HIP106	VHVASGY	7	NA	>2000	μM
HIP133	PAETGQET	8	NA	>2000	μM
HIP89	GRWPVKT	7	NA	>2000	μM
HIP127	HTDNGSNF	8	NA	>2000	μM
HIP199	TAYALLKLAGRW	12	NA	>333	μM
HIP201	TAYFLAKLAGRW	12	NA	>333	μM
HIP204	TAYFLLKAAGRW	12	NA	>333	μM
HIP207	TAYFLLKLAGRA	12	NA	>333	μM
HIP161	ACWGAGIKQEF	11	NA	>333	μM
HIP171	ACWWAGIRQEF	11	NA	>333	μM
HIP166	ACWWAGIKQAF	11	NA	>333	μM
HIP163	ACWWAGAKQEF	11	NA	>333	μM
HIP165	ACWWAGIKAEF	11	NA	>333	μM
HIP160	ACWAAGIKQEF	11	NA	>333	μM
HIP179	AAYFLLKLAGRW	12	NA	100±10	μM
HIP202	TAYFLLALAGRW	12	NA	113±15	μM

HIP200	TAYFALKLAGRW	12	NA	115±21	μM
HIP205	TAYFLLKLAARW	12	NA	118±10	μM
HIP198	TASFLLKLAGRW	12	NA	186±23	μM
HIP197	TAAFLLKLAGRW	12	NA	193±10	μM
HIP209	TAYFLLKLAGRW	12	NA	21±7	μM
HIP167	ACWWAGIKQEA	11	NA	245±13	μM
HIP157	AAWWAGIKQEF	11	NA	277±47	μM
HIP174	ASWWAGIKQEF	11	NA	294±41	μM
HIP208	TAYFLLKLAGRL	12	NA	315±30	μM
HIP158	ACAWAGIKQEF	11	NA	33±6	μM
HIP203	TAYFLLILAGRW	12	NA	4.1±0.7	μM
HIP159	ACGWAGIKQEF	11	NA	46±5	μM
HIP164	ACWWAGIAQEF	11	NA	62±13	μM
HIP210	TAYFLLKLAGRW	12	NA	65±8	μM
HIP206	TAYFLLKLAGAW	12	NA	83±15	μM
HIP162	ACWWAAIKQEF	11	NA	90±10	μM
HIP168	ACWWAGIKQEF	11	NA	95±9	μM
HIP1147	ILPWKWPWWPWPP	13	NA	16	μM
HIP1145	HCKFWW	6	NA	90	μM
HIP308	IRILQQLLFIHFRIG	15	NA	1.3	μM
HIP368	VEAIIRILQQLLFIH	15	NA	7.8	μM
HIP345	QQLLFIHFRIGCQHS	15	NA	76	μM
HIP300	HFPRIWLHSLGQHIY	15	NA	187	μM
HIP292	FIHFRIGCQHSRIGI	15	NA	>>200	μM
HIP362	TWAGVEAIIRILQQL	15	NA	>>201	μM
HIP1165	DLHTHAQ	7	NA	30	μM
HIP231	YQLLIRMIYKNI	12	HeLa	5	μM
HIP148	YQLLIRMIY	9	HeLa	6	μM
HIP229	YQLLIRMIYKAI	12	HeLa	7	μM
HIP218	YALLIRMIYKNI	12	HeLa	8	μM
HIP777	QLLIRMIYKNILFYLVPGPGHGAEPERRNIKYL	33	HeLa	9	μM
HIP230	YQLLIRMIYKNA	12	HeLa	11	μM
HIP228	YQLLIRMIYANI	12	HeLa	12	μM
HIP222	YQLAIRMIYKNI	12	HeLa	14	μM
HIP178	QLLIRMIYKNI	11	HeLa	21	μM
HIP224	YQLLIAMIYKNI	12	HeLa	34	μM
HIP226	YQLLIRMAYKNI	12	HeLa	35	μM

HIP227	YQLLIRMIAKNI	12	HeLa	40	µM
HIP223	YQLLARMIYKNI	12	HeLa	45	µM
HIP173	AEPERRNIKYL	11	HeLa	50	µM
HIP225	YQLLIRAIYKNI	12	HeLa	70	µM
HIP770	RMIYKNILFYLVPGPGHGAEPERRNIKYL	29	HeLa	85	µM
HIP138	YQLLIRMI	8	HeLa	120	µM
HIP221	YQALIRMIYKNI	12	HeLa	165	µM
HIP776	LSELDDRADALQAGASQFETSAAKLKRK YWWKN	33	HeLa	>200	µM
HIP154	LFYLVPGPGH	10	HeLa	>200	µM
HIP98	QLLIRMI	7	HeLa	>200	µM
HIP232	YQLLIRPIYKNI	12	HeLa	>200	µM
HIP114 6	WQCLTLTHRGFVLLTITVLR	20	HeLa	12	µM
HIP113 9	IHAEIKNSLKIDNLDVNRCIEAL	23	NA	25	µM
HIP000	FHNHGKQ	7	-	-	-
HIP00N	FHNHAKQ	7	-	-	-

Table S2. Binding scores of 80 peptides against the HIV-1 integrase protein obtained from HADDOCK.

Peptide ID	Haddock Score
HIP1142	-226.5 +/- 16.9
HIP678	-190.8 +/- 16.8
HIP776	-184.5 +/- 28.0
HIP1113	-184.5 +/- 10.1
HIP1140	-183.0 +/- 9.0
HIP777	-181.2 +/- 10.8
HIP404	-175.8 +/- 12.9
HIP1133	-168.6 +/- 9.3
HIP1134	-166.4 +/- 2.8
HIP161	-163.4 +/- 2.6
HIP150	-162.5 +/- 8.8
HIP166	-162.0 +/- 7.7
HIP1135	-159.0 +/- 17.1
HIP170	-158.5 +/- 3.7
HIP1143	-158.2 +/- 3.9
HIP288	-157.9 +/- 10.8

HIP153	-157.6 +/- 15.6
HIP1144	-155.9 +/- 26.9
HIP155	-155.2 +/- 7.6
HIP271	-154.9 +/- 10.1
HIP1114	-154.0 +/- 5.9
HIP1146	-151.7 +/- 6.6
HIP160	-151.1 +/- 12.4
HIP1059	-147.3 +/- 9.9
HIP56	-144.6 +/- 10.2
HIP1136	-143.9 +/- 12.8
HIP300	-141.5 +/- 18.2
HIP1137	-140.3 +/- 20.4
HIP345	-139.4 +/- 9.2
HIP368	-139.1 +/- 9.2
HIP1165	-137.7 +/- 1.3
HIP173	-137.5 +/- 3.8
HIP200	-137.2 +/- 2.9
HIP1115	-136.7 +/- 6.2
HIP1138	-136.3 +/- 7.8
HIP26	-136.1 +/- 7.5
HIP75	-135.6 +/- 5.8
HIP127	-135.4 +/- 8.6
HIP1060	-134.7 +/- 7.7
HIP35	-134.7 +/- 5.3
HIP201	-134.5 +/- 6.5
HIP202	-133.7 +/- 6.3
HIP30	-132.6 +/- 4.6
HIP178	-132.3 +/- 5.2
HIP308	-131.4 +/- 1.7
HIP227	-130.8 +/- 3.9
HIP38	-130.7 +/- 7.3
HIP223	-129.4 +/- 1.7
HIP149	-128.7 +/- 7.6
HIP156	-128.0 +/- 1.0
HIP36	-127.5 +/- 4.8
HIP204	-126.5 +/- 6.0
HIP232	-125.9 +/- 5.6
HIP152	-124.8 +/- 0.4
HIP292	-123.5 +/- 13.8

HIP221	-122.1 +/- 6.3
HIP31	-121.8 +/- 5.4
HIP226	-121.8 +/- 2.3
HIP224	-121.5 +/- 6.9
HIP84	-121.4 +/- 6.8
HIP230	-120.2 +/- 7.6
HIP199	-119.0 +/- 3.9
HIP81	-117.5 +/- 3.8
HIP231	-117.5 +/- 2.7
HIP362	-116.5 +/- 11.0
HIP225	-116.0 +/- 2.7
HIP1141	-115.8 +/- 10.0
HIP37	-115.5 +/- 11.0
HIP151	-114.7 +/- 1.0
HIP218	-113.8 +/- 4.4
HIP154	-112.9 +/- 11.3
HIP207	-109.7 +/- 7.1
HIP148	-109.0 +/- 3.2
HIP138	-106.8 +/- 4.7
HIP1128	-105.1 +/- 5.3
HIP106	-101.5 +/- 4.4
HIP89	-99.0 +/- 2.4
HIP88	-98.5 +/- 1.7
HIP87	-93.8 +/- 1.6
HIP133	-55.0 +/- 7.7
HIP000	**-119.0 +/- 2.5**
HIP00N	**-119.2 +/- 4.1**

Table S3: Non-bonding interaction of best 6 docking complexes with control peptide.

Peptide ID (Docking Score)	Protein residues	Bond Distance (Å)	Interaction Category
**HIP000**	GLU92	5.02821	Electrostatic
**(-119.0 +/- 2.5)**	ASP116	5.05949	Electrostatic
	GLU92	2.34612	Hydrogen Bond
	GLN148	2.84401	Hydrogen Bond
	MG	1.69451	Other
	SER147	2.95941	Other
	HIS67	3.99072	Hydrophobic
Peptide Sequence (HIP000): FHNHGKQ			

**HIP1113**<br>**(-184.5 +/- 10.1)**	LYS101	1.6305	Hydrogen Bond; Electrostatic
	LYS101	4.64576	Electrostatic
	GLU97	4.6439	Electrostatic
	GLU102	5.16261	Electrostatic
	ASP61	3.9002	Electrostatic
	GLU37	2.51884	Hydrogen Bond
	GLN93	2.6772	Hydrogen Bond
	GLU97	2.13476	Hydrogen Bond
	GLU97	1.62029	Hydrogen Bond
	ASP61	2.33918	Hydrogen Bond
	ASN62	2.02467	Hydrogen Bond
	ASN100	2.72819	Hydrogen Bond
	SER92	2.53619	Hydrogen Bond
	GLN93	2.64715	Hydrogen Bond
	ASP61	2.75662	Hydrogen Bond
	VAL95	3.85929	Hydrophobic
	HIS12	5.09799	Hydrophobic
	LYS101	4.90704	Hydrophobic
Peptide Sequence:	DQAEHLKTAVQMAVFIHNYKA		
**HIP1140**<br>**(-183.0 +/- 9.0)**	LYS159	1.62763	Hydrogen Bond;Electrostatic
	LYS159	1.58299	Hydrogen Bond;Electrostatic
	MG	3.72705	Electrostatic
	SER119	1.73887	Hydrogen Bond
	SER119	1.68291	Hydrogen Bond
	ASN155	2.36372	Hydrogen Bond
	GLY149	2.59481	Hydrogen Bond
	GLU152	1.79353	Hydrogen Bond
	HIS67	2.90708	Hydrogen Bond
	GLY118	2.10135	Hydrogen Bond
	LYS156	2.24905	Hydrogen Bond
	LYS159	2.70634	Hydrogen Bond
	SER147	2.94203	Hydrogen Bond
	GLN148	1.99581	Hydrogen Bond
	GLU152	2.782	Hydrogen Bond
	MG	1.68456	Other
	HIS67	4.47377	Hydrophobic
	HIS67	5.43175	Hydrophobic
Peptide Sequence:	PDIVIYQYMDDLYVGSDLEI		
**HIP1142**<br>**(-226.5 +/- 16.9)**	LYS156	5.43345	Electrostatic
	MG	3.83883	Electrostatic
	MG	1.52159	Electrostatic;Other

	MG	1.53182	Electrostatic;Other
	MG	1.58749	Electrostatic;Other
	ASP116	4.77462	Electrostatic
	GLN148	2.90661	Hydrogen Bond
	SER147	1.78528	Hydrogen Bond
	GLN148	1.75637	Hydrogen Bond
	GLY118	2.51929	Hydrogen Bond
	GLN148	2.46063	Hydrogen Bond
	HIS67	2.87898	Hydrophobic
	VAL150	4.82377	Hydrophobic
	VAL150	5.32	Hydrophobic
	LEU68	4.99629	Hydrophobic
	VAL150	5.18276	Hydrophobic
	VAL150	4.535	Hydrophobic
	LYS156	5.28863	Hydrophobic
Peptide Sequence:	ETWETWWTEYWQATWIPEWE		
**HIP678 (-190.8 +/- 16.8)**	LYS159	1.64281	Hydrogen Bond;Electrostatic
	MG	4.49734	Electrostatic
	MG	1.59752	Electrostatic;Other
	MG	1.57005	Electrostatic;Other
	MG	1.54703	Electrostatic;Other
	HIS67	1.85516	Hydrogen Bond
	SER119	2.13765	Hydrogen Bond
	SER119	2.8161	Hydrogen Bond
	SER119	2.10608	Hydrogen Bond
	ASN120	2.06765	Hydrogen Bond
	ASN155	1.96797	Hydrogen Bond
	ASN155	2.55117	Hydrogen Bond
	GLY149	2.16305	Hydrogen Bond
	THR122	2.15001	Hydrogen Bond
	THR122	1.90324	Hydrogen Bond
	SER119	1.68793	Hydrogen Bond
	THR66	2.4754	Hydrogen Bond
	GLY118	2.18086	Hydrogen Bond
	GLY118	2.24255	Hydrogen Bond
	SER119	2.51189	Hydrogen Bond
	SER119	2.05558	Hydrogen Bond
	GLY123	2.72866	Hydrogen Bond
	GLY149	2.54727	Hydrogen Bond
	GLU152	2.64034	Hydrogen Bond
	ASN155	2.39868	Hydrogen Bond
	THR122	2.22941	Hydrogen Bond

	ALA91	4.68285	Hydrophobic
	LYS156	4.95702	Hydrophobic
Peptide Sequence:	PDIVIYQYMDDLYVGSDLEI		
**HIP776 (-184.5 +/- 28.0)**	GLU152	1.61204	Hydrogen Bond;Electrostatic
	MG	3.85234	Electrostatic
	MG	1.62773	Electrostatic
	HIS67	2.86958	Hydrogen Bond
	ASN120	2.47947	Hydrogen Bond
	ASN155	2.143	Hydrogen Bond
	LYS156	1.80561	Hydrogen Bond
	LYS156	1.81255	Hydrogen Bond
	LYS159	1.86094	Hydrogen Bond
	TYR143	1.80587	Hydrogen Bond
	ASN155	2.57454	Hydrogen Bond
	LYS156	2.59427	Hydrogen Bond
	HIS67	2.7525	Hydrogen Bond
	GLY149	2.80079	Hydrogen Bond
	LYS159	2.81587	Hydrogen Bond
	GLY163	2.54644	Hydrogen Bond
	GLN148	2.44438	Hydrogen Bond
	GLU152	2.98254	Hydrogen Bond
	ASN155	2.6971	Hydrogen Bond
	LYS156	2.8897	Hydrogen Bond
	LYS156	4.80763	Hydrophobic
	VAL150	4.47809	Hydrophobic
	LYS159	3.93831	Hydrophobic
	HIS67	5.36223	Hydrophobic
	LYS156	4.54612	Hydrophobic
	LYS160	5.13022	Hydrophobic
Peptide Sequence:	LSELDDRADALQAGASQFETSAAKLKRKYWWKN		
**HIP777 (-181.2 +/- 10.8)**	GLU69	1.6365	Hydrogen Bond;Electrostatic
	ASP64	1.81943	Hydrogen Bond;Electrostatic
	ASP64	1.5773	Hydrogen Bond;Electrostatic
	MG	1.55919	Electrostatic;Other
	MG	4.85895	Electrostatic
	CYS65	2.42435	Hydrogen Bond
	CYS65	2.37602	Hydrogen Bond
	GLU69	1.87098	Hydrogen Bond
	TYR143	1.70553	Hydrogen Bond
	GLN148	1.84263	Hydrogen Bond
	LYS156	1.98758	Hydrogen Bond

	LYS156	2.05643	Hydrogen Bond
	LYS159	2.44466	Hydrogen Bond
	LYS159	2.11668	Hydrogen Bond
	PRO145	2.71943	Hydrogen Bond
	GLN146	1.95072	Hydrogen Bond
	SER147	2.74621	Hydrogen Bond
	TYR143	2.35739	Hydrogen Bond
	GLN148	1.70363	Hydrogen Bond
	ASN155	2.2906	Hydrogen Bond
	ASN155	2.65175	Hydrogen Bond
	GLY149	2.69496	Hydrogen Bond
	CYS65	2.9663	Hydrogen Bond
	ASN155	1.79245	Hydrogen Bond
	LEU68	2.12997	Hydrogen Bond
	LYS156	2.62386	Hydrogen Bond
	LYS159	2.33698	Hydrogen Bond
	GLN148	2.84709	Hydrogen Bond
	GLN146	2.32899	Hydrogen Bond
	LYS160	2.79932	Hydrogen Bond
	HIS67	2.09851	Hydrogen Bond
	TYR143	5.11194	Hydrophobic
	ASN155,LYS156	4.65934	Hydrophobic
	VAL150	5.33929	Hydrophobic
	LYS159	3.91548	Hydrophobic
	LYS160	5.45756	Hydrophobic
	LYS160	5.37835	Hydrophobic
	HIS67	4.72697	Hydrophobic
	LYS156	4.43536	Hydrophobic
	LYS159	5.49932	Hydrophobic
	ARG166	5.27362	Hydrophobic
Peptide Sequence:	QLLIRMIYKNILFYLVPGPGHGAEPERRNIKYL		

Table S4. Stepwise multiple linear regression with most relevant peptide properties as predictors of binding affinity (HADDOCK score) of top 30 peptides with the HIV integrase protein.

	**Step 1 (R^2=0.74)**	**Step 2 (R^2=0.74)**	**Step 3 (R^2=0.74)**	**Step 4 (R^2=0.74)**	**Step 5 (R^2=0.73)**	**Step 6 (R^2=0.68)**	**Step 7 (R^2=0.74)**
**Predictors of FireDock score**	**P-Values**						
Aromatic AA	0.0285	0.0042	0.0034	0.0008	0.0003	0.0018	0.0025
Vol (A^3)	0.4582	0.2215	0.1433	0.0272	0.0023	0.0101	0.0010

Net charge at PH 7	0.4628	0.4289	0.3012	0.3077	0.0005	0.0003	0.0014
Extinction coefficient	0.0157	0.0095	0.0067	0.0056	0.0069	**0.0599**	
Nonpolar AA	0.4234	0.3052	0.1211	0.0455	**0.0430**		
Basic AA (+ve)	0.5321	0.4628	0.5296	**0.3910**			
Polar AA	0.8031	0.6533	**0.7822**				
Gravy	0.6646	**0.6605**					
Acidic AA (-ve)	**0.9345**						

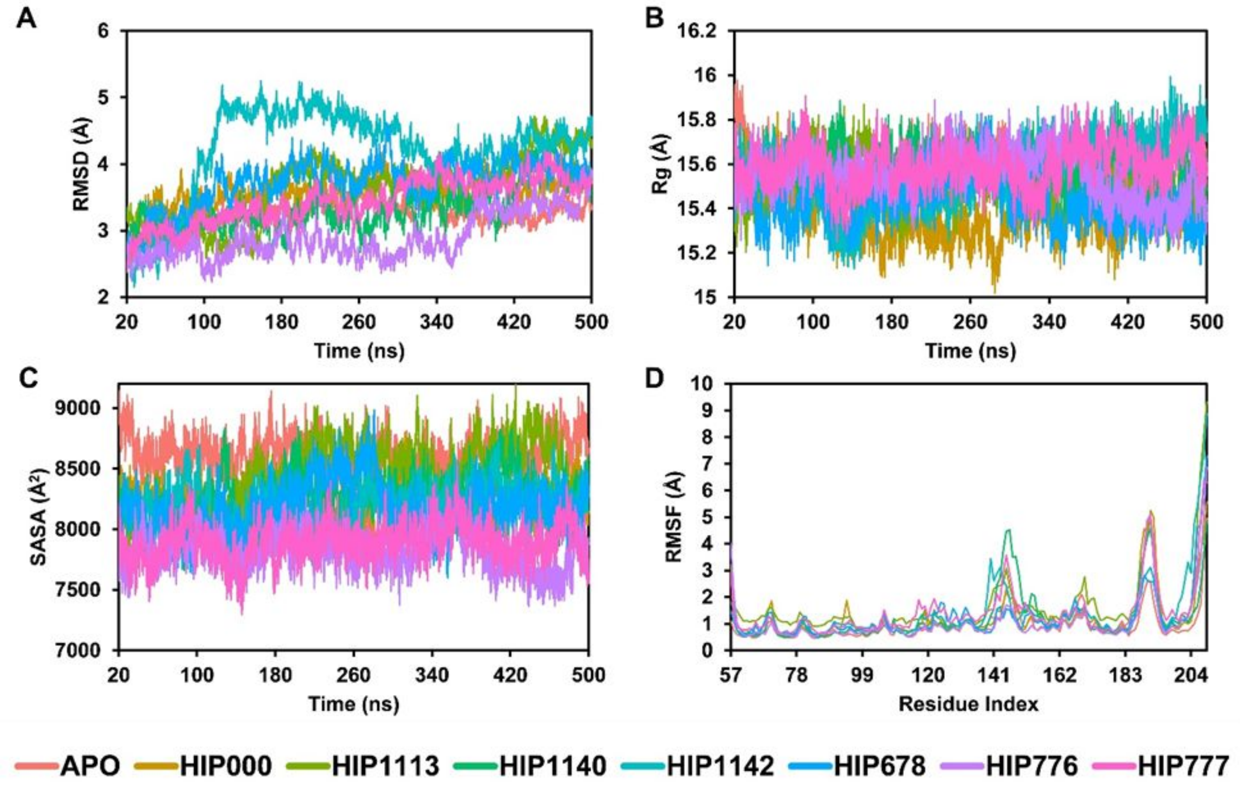

Figure S1. Molecular dynamics simulation of seven peptide-integrase complexes. (A) Root mean squared deviation (RMSD) of α carbon; (B) Radius of gyration (Rg); (C) Solvent accessible surface area (SASA); (D) Root mean squared fluctuation (RMSF); [Initial 20 ns RMSD, Rg, and SASA were considered as stabilization period].

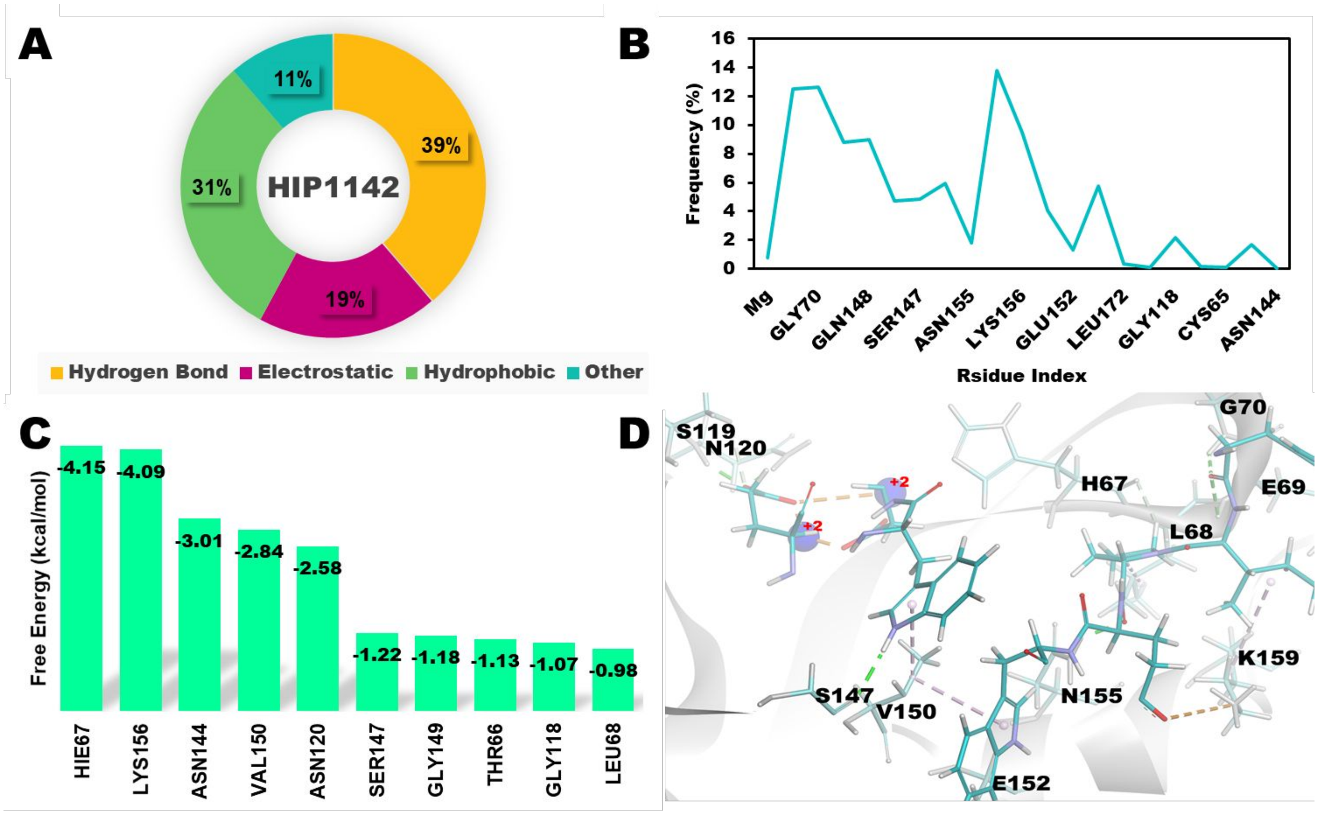

Figure S2. Integrase-HIP1142 complex. (A) Distribution of non-covalent interactions; **(B)** Interacting residues of HIP1142; **(C)** Free energy distribution of top ten residues; **(D)** Representative snapshot at 1000th ns: IN (grey), HIP1142 (Deep Sky Blue) & Mg^{2+} (blue).

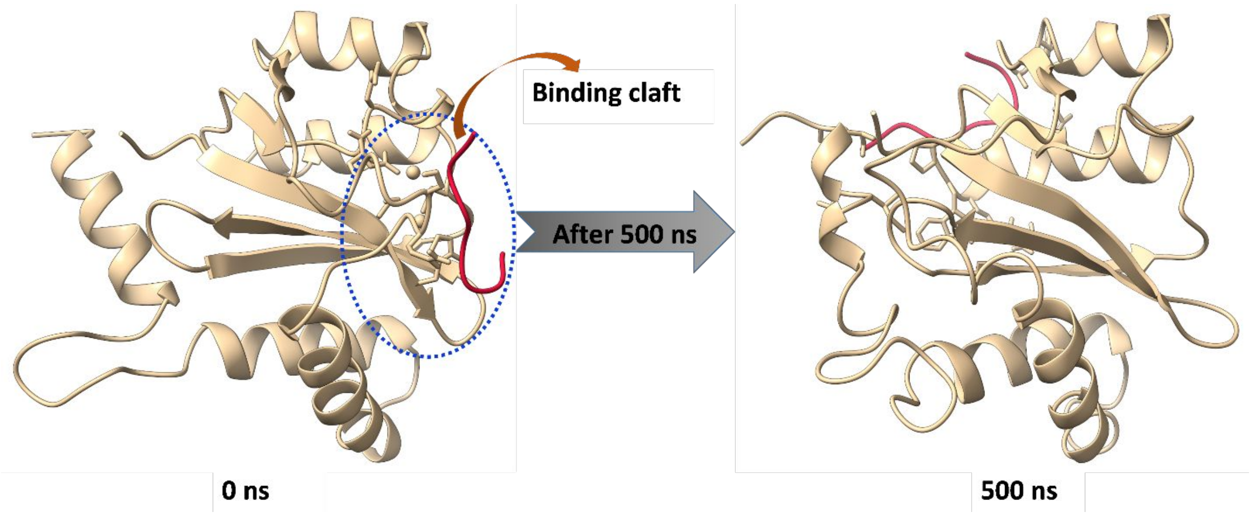

Figure S3: Integrase-HIP00N complex. Binding pose at 0 ns and 500 ns after simulation peptide shifted from its initial position to a new position.

Negative Control.mp4

Trajectory movie SM1: Provide a clear view of deviation of negative control peptide from the binding pocket.

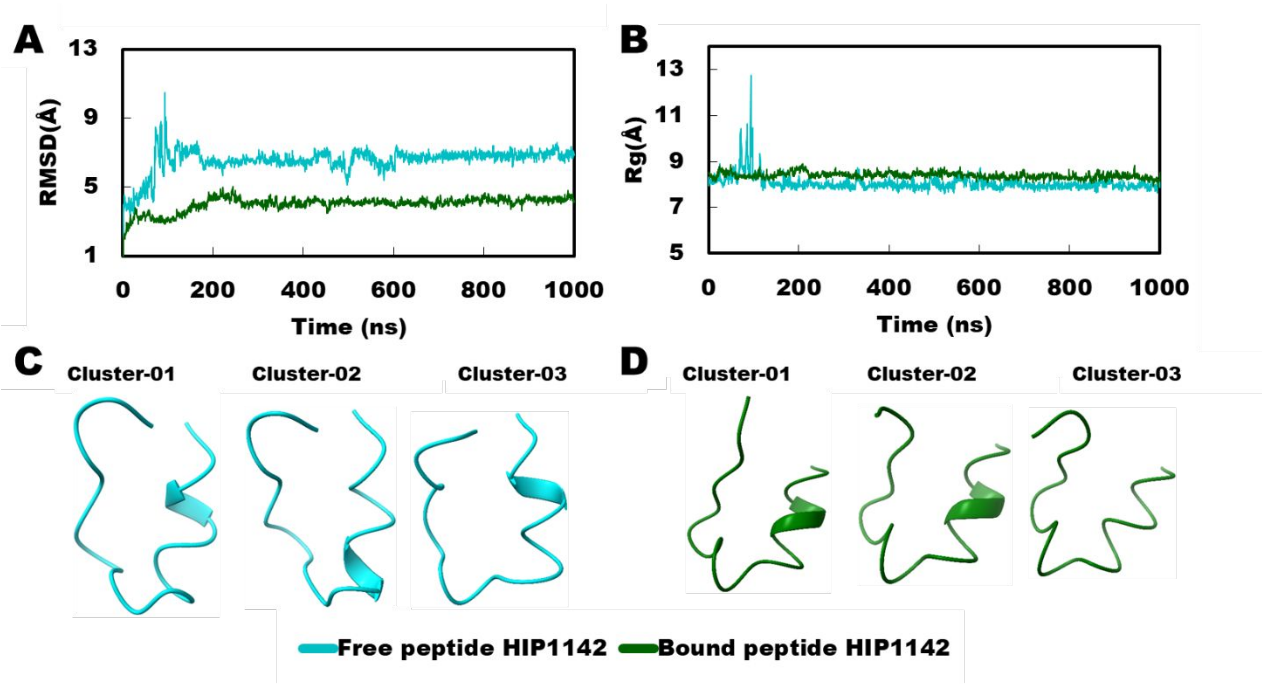

Figure S4. Comparison of HIP776 peptide conformation on free and bound state. (A) RMSD in free and bound state; (B) Rg in free and bound state; (C) Best three trajectory clusters in free state; (D) Best three trajectory clusters in bound state.

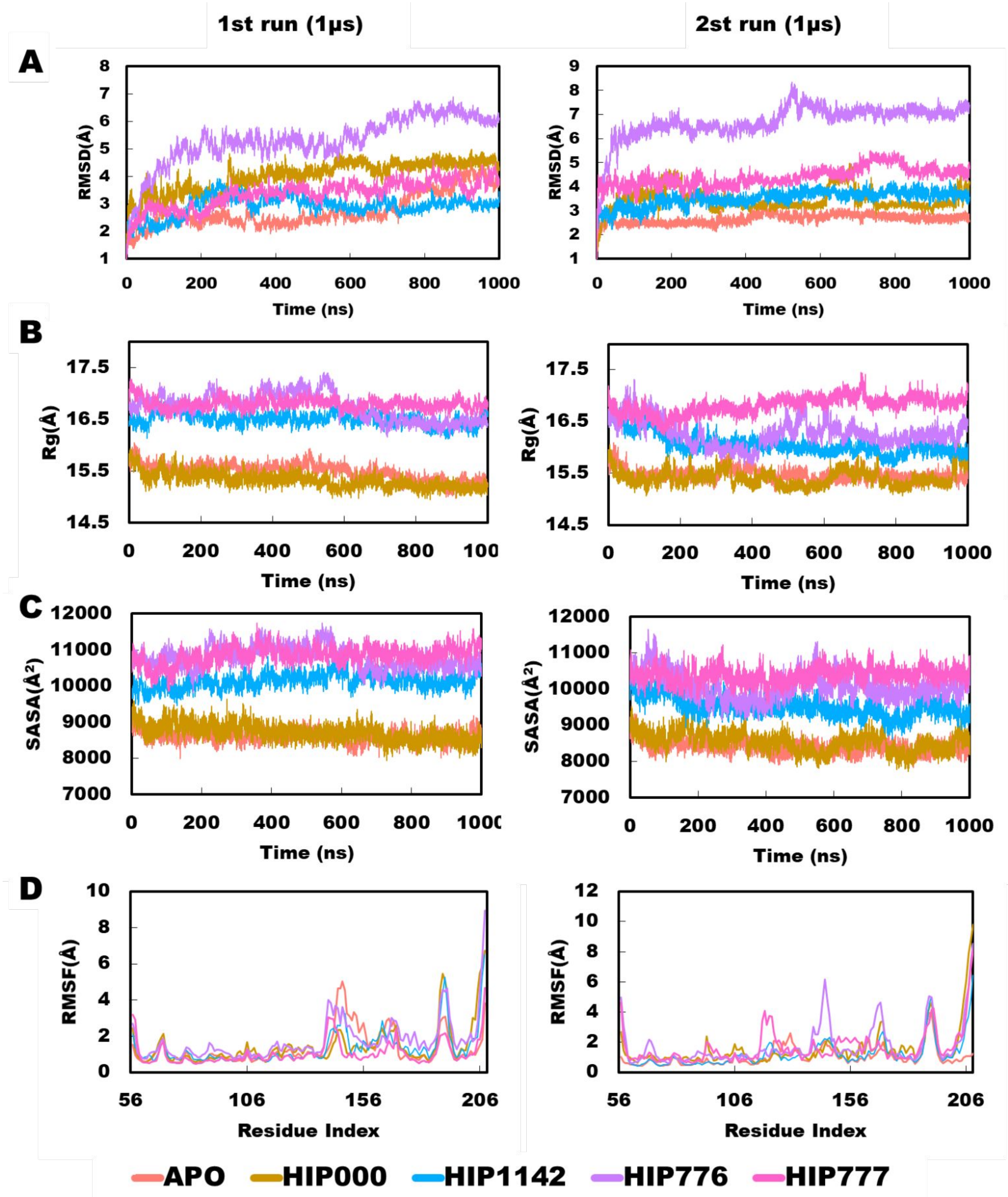

Figure S5. Comparison of simulation analysis variables used in this study between two independent simulation run. (A) RMSD; (B) Rg; (C) SASA, (D) RMSF
